# Profiling the Atlantic Salmon IgM^+^ B Cell Surface Proteome: Novel Information on Teleost Fish B Cell Protein Repertoire and Identification of Potential B Cell Markers

**DOI:** 10.3389/fimmu.2019.00037

**Published:** 2019-01-29

**Authors:** Ma. Michelle D. Peñaranda, Ingvill Jensen, Linn G. Tollersrud, Jack-Ansgar Bruun, Jorunn B. Jørgensen

**Affiliations:** ^1^The Norwegian College of Fishery Science, Faculty of Biosciences, Fisheries and Economics, UiT The Arctic University of Norway, Tromsø, Norway; ^2^Tromsø University Proteomics Platform, Institute of Medical Biology, UiT The Arctic University of Norway, Tromsø, Norway

**Keywords:** B cells, cell surface markers, teleost fish, salmon, CD22, CD79A, IgM, proteomics

## Abstract

Fish immunology research is at a pivotal point with the increasing availability of functional immunoassays and major advances in *omics* approaches. However, studies on fish B cells and their distinct subsets remain a challenge due to the limited availability of differentially expressed surface markers. To address this constraint, cell surface proteome of Atlantic salmon IgM^+^ B cells were analyzed by mass spectrometry and compared to surface proteins detected from two adherent salmon head kidney cell lines, ASK and SSP-9. Out of 21 cluster of differentiation (CD) molecules identified on salmon IgM^+^ B cells, CD22 and CD79A were shortlisted as potential markers based on the reported B cell-specific surface expression of their mammalian homologs. Subsequent RT-qPCR analyses of flow cytometry-sorted subpopulations from head kidney leukocytes confirmed that both *cd22* and *cd79a* genes were highly expressed in IgM^+^ lymphoid cells but were observed in barely detectable levels in IgM^−^ non-lymphoid suspension and adherent cells. Similarly, significantly high *cd22* and *cd79a* mRNA levels were observed in IgM^+^ or IgT^+^ lymphoid cells from the spleen and peritoneal cavity, but not in their corresponding IgM^−^ IgT^−^ non-lymphoid fractions. This suggests that the B cell restrictive expression of CD22 and CD79A extend down to the transcription level, which was consistent across different lymphoid compartments and immunoglobulin isotypes, thus strongly supporting the potential of CD22 and CD79A as pan-B cell markers for salmon. In addition, this study provides novel information on the salmon B cell surface protein repertoire, as well as insights on B cell evolution. Further investigation of the identified salmon CD molecules, including development of immunological tools for detection, will help advance our understanding of the dynamics of salmon B cell responses such as during infection, vaccination, or immunostimulation.

## Introduction

The sustainability of aquaculture is constantly being challenged by the occurrence and re-occurrence of infectious diseases (1). Fish vaccination has become the main prophylactic strategy against these economically-devastating pathogens. However, unlike many bacterial vaccines that are highly protective, most of the available vaccines against viral pathogens in salmon only provide suboptimal protection (2). It is not clear why the elicited immune responses of fish virus vaccines are not efficient in providing protection against subsequent infection. Consequently, an important question is: What set of host responses constitute protective immunity in salmon? Critical to this host response are the B cells with diverse functional properties that encompass both the innate and adaptive arms of the immune system, including antigen presentation (3), phagocytosis (4), production of natural (5) and antigen-specific antibodies (Abs) (6, 7), and the generation of immunological memory [reviewed in (8)].

Different lineages and subsets of B cells exist, each exhibiting specific phenotypic characteristics that respond differentially to TLR ligands, pathogens, and/or immunogens. In mammals, four subsets that belong either to the B-1 or B-2 lineages have been clearly defined. B-2 cells consist of two subpopulations: the more conventional follicular (FO) B cells that constitute the major subset in the spleen and trigger the formation of germinal centers and the subsequent production of plasmablasts, plasma cells, and memory B cells with high affinity Ag-binding capacities upon activation of T cell dependent (TD) antigens [reviewed in (9)]; and the marginal zone (MZ) B cells that integrate classical innate and adaptive signaling pathways to mount rapid antibody responses, particularly to blood-borne pathogens [reviewed in (10)]. Similar to MZ B cells, B-1 cells, which can be subdivided further into B1a and B1b based on their CD5 surface expression, have important innate functions such as phagocytosis and production of polyreactive natural Abs in a T cell independent (TI) manner (11, 12). The B-1 cells are predominantly located in the peritoneal cavity (13), but are also present in the spleen and other lymphoid organs at very low levels. While initially thought to lack memory B cell generation, recent data have shown that these innate-like B cells also generate memory B cells during TI immune responses [reviewed in (14–16)].

As the first vertebrate group that possesses all elements of adaptive immunity, teleost fish are able to execute immune functions comparable to that of mammals. Although clear differences exist between the structure and organization of the teleost and mammalian immune systems, functional equivalent lymphoid compartments have been reported [reviewed in (17, 18)]. Analogous to the mammalian bone marrow (BM), the teleost head kidney (HK) serves as both the major hematopoietic tissue and reservoir for long-lived plasma cells (6). In the absence of lymph nodes, teleost spleen constitutes as the main secondary lymphoid organ, where the majority of naïve B cells mature and circulate for continuous immune surveillance. In addition to the systemic lymphoid compartments, teleost peritoneal cavity also houses B cells whose development and migration pathways remain largely unexplored (19). In contrast to mammals, however, teleosts lack follicular structures and do not form distinguishable germinal centers (20). Little is still also known about the characteristics of teleost memory B cells but it appears to have a relatively low proliferation potential (6). Moreover, their systemic Ab responses rely on unswitched low-affinity IgM responses (21).

Three classes of immunoglobulins (Igs) have been identified in teleosts: IgM, IgD, and IgT (or IgZ in some species), with IgM^+^ being the predominant surface Ig isotype (22). IgD is usually co-expressed on the surface of teleost IgM^+^ B cells, although single positive IgM^+^ or IgD^+^ B cells (23) also exist. IgT^+^ only B cells comprise a separate lineage of fish B cells that appears to have a main role in mucosal immunity (24). Morphological and functional studies suggest that teleost B cells resemble mammalian B1 cells more than B2 cells (25, 26). In fact, it is hypothesized that mammalian innate-like B cells, characterized by high surface IgM expression (27), evolved from fish IgM^+^ B cells (18), with the B2 lineage emerging later as a more efficient subset that gradually acquired a dominant role in the mammalian adaptive immunity. Fish B cells exhibit both innate and adaptive immune functions (6), but whether these functions are performed by distinct B cell subsets or not is unknown. Specifically, which B cell subpopulation/s and/or lineages play an important role in protection against infection and/or immunity following vaccination are still open questions in fish immunology.

Cluster of differentiation (CD) is a system used for identifying cell surface markers for various leukocyte subpopulations, including B cells. At present, at least 371 CD proteins have been reported in mammals (28)—making immunophenotyping a rather trivial task. In contrast to the mammalian system where different lineages and subtypes of B cells can be identified and sorted with greater clarity through commercially available marker Abs, studying the dynamics of fish B cell responses has been challenging due to lack of pan and subset-specific markers. For Atlantic salmon, in particular, B cells are currently sorted from the total leukocyte population using surface Igs as sole markers, typically via the predominant IgM isotype (22, 29). While this approach has been extremely useful, the binding of Abs to surface Igs could trigger unwanted activation of the BCR, which may interfere with downstream assays. In addition, since the status of surface Ig expression of salmon B cells at various stages of differentiation (i.e., putative naïve B cells, plasmablasts, plasma cells, or memory B cells) is largely unknown, some of these subsets may not be detected during sorting and hence, will be excluded from further analysis.

To address this current limitation, we aimed to identify CD molecules than can be potentially used as pan- or subset-specific B cell markers and, in turn, facilitate molecular, and functional investigations of the heterogeneous salmon B cell population. Additionally, we aimed to profile the salmon B cell surface proteome in order to have a better understanding of the phenotypic characteristics of teleost IgM^+^ B cells.

## Materials and Methods

### Experimental Fish

Healthy Atlantic salmon (*Salmo salar* L.) QTL fish strain Aquagen standard (Aquagen, Kyrksæterøra, Norway) were obtained from the Tromsø Aquaculture Research Station (Tromsø, Norway). Fish were kept at 10°C in tanks supplied with running filtered water, natural light and fed on commercial dry feeds (Skretting, Stavanger, Norway). Estimated weight of fish used for isolation of peripheral blood leukocytes (PBL) and subsequent sorting of IgM^+^ B cells for proteomics analyses was 700–900 g. Head kidney leukocytes (HKL) were collected separately from the same batch of fish. Peritoneal cavity leukocytes (PeL) and splenocytes (SpL) were collected simultaneously from another batch of smaller fish (estimated mean weight: ~60 g).

### Cell Culture

Atlantic Salmon Kidney (ASK) cells (30) and *Salmo salar* pronephros 9 (SSP-9) cells (31), derived from the major hematopietic tissue of Atlantic salmon, were grown as monolayers at 20°C in Leibovitz (L-15) medium (Gibco, Life Technologies). ASK cell culture medium was supplemented with P/S (100 units/mL penicillin, 100 μg/mL streptomycin) and 12% fetal bovine serum (FBS), while SSP-9 cell culture medium was supplemented with 50 μg/mL gentamycin and 8% FBS. Five T-75 flasks were seeded with ASK or SSP-9 cells at a density of ~2 × 10^6^ cells per flask and collected after 72 h at 90% confluence for subsequent cell surface protein isolation.

### Tissue Collection and Leukocyte Isolation

Blood was extracted from the caudal vein of Atlantic salmon using a vacutainer with 68 I.U. sodium heparin (Becton Dickinson) and immediately transferred into transport medium (L-15 with P/S, 2% FBS, and 20 IE/mL heparin). Spleen and HK were aseptically collected into transport medium after ensuring that all blood was drained from fish tissues. Cells from salmon peritoneal cavity were obtained by lavage and immediately stored in transport medium.

Leukocyte isolations (PBL, HKL, SpL, or PeL) were performed on Percoll gradients as described previously (32). Blood suspension was placed directly onto 54% Percoll (GE Healthcare) and centrifuged at 400 × g for 40 min at 4°C. Spleen and HK were homogenized on 100-μm cell strainers (Falcon), loaded onto 25/54% discontinuous Percoll gradients, and centrifuged as above. Similarly, peritoneal cavity cells were loaded onto 25/54% discontinuous Percoll gradient for PeL isolation. Leukocytes at the interface were collected and washed twice in L-15 with P/S before further use.

For stimulation with lipopolysaccharide (LPS), freshly isolated PBLs were seeded in two T25 flasks (Nunclon Delta Surface ThermoFisher Scientific, 6.25 × 10^6^ cells/flask). One flask was treated with 50 μg/mL LPS (purified by Phenol extraction from *Escherichia coli* O111:B4, Sigma-Aldrich) diluted in Dulbecco's Phosphate Buffered Saline (DPBS; Sigma-Aldrich), while control group received only DPBS. Cells were incubated at 14°C for 72 h before staining, sorting, and surface protein isolation as detailed below.

### Cell Staining and FACS Sorting

Total leukocytes were centrifuged at 500 × g, resuspended in PBS^+^ (Dulbecco PBS with 1% BSA, filter-sterilized), and stained with anti-salmon IgM (IgF1-18) (1:200 dilution) and/or anti-trout IgT (2 μg/mL) monoclonal antibodies (mAbs) for 30 min. These mAbs were generously provided by Dr. Karsten Skjødt and Prof. Oriol Sunyer, respectively. Salmon anti-IgM have been shown to recognize both IgM-A and -B isotypes of Atlantic salmon (29), while trout α-IgT has been previously validated for cross-specificity with Atlantic salmon IgT (22). After two washing steps, leukocytes were incubated with isotype specific secondary Abs: IgG1-RPE (1:400 dilution) and IgG2a-APC (1:400 dilution), respectively, and viability dye FVD780 (1 μL/mL; eBioscience) in PBS^+^ for 20 min. All staining and centrifugation steps were done at 4°C.

**Figure 1:**
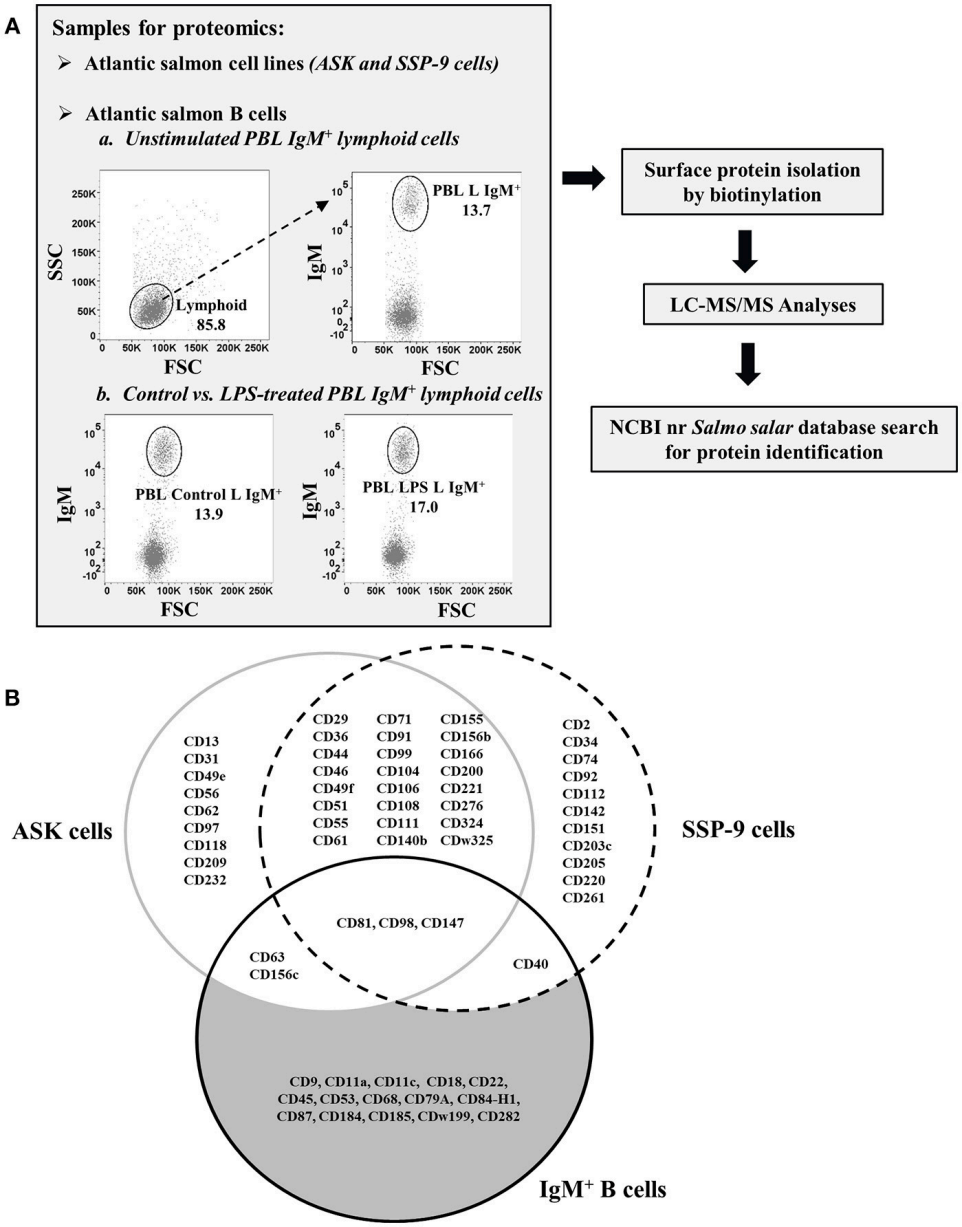
Isolation and identification of CD molecules on surface of Atlantic salmon B cells and head kidney cell lines. **(A)** Schematic diagram of proteomics workflow performed in the study. **(B)** Venn diagram comparing the CD molecules detected on cell surface of sorted IgM^+^ PBLs vs. head kidney cell lines, ASK and SSP-9, of Atlantic salmon.

Stained leukocytes were resuspended in PBS^+^ at 5.0 × 10^7^ cells/mL for sorting using the BD FACS Aria III flow cytometer (BD Biosciences). Dead cells (FVD780^+^) and doublets (SSC-A vs. SSC-H) were excluded from the population. Remaining cells were sorted on the basis of their forward scatter (FSC) and side scatter (SSC) profiles, and then on their IgM^+^ (RPE fluorescence emission) and/or IgT^+^ (APC fluorescence emission) surface expression. FSC^low^ SSC^low^ subpopulation that excludes granulocytes and myeloid cells was designated lymphoid gate. Cells outside this gate were considered “non-lymphoid” (nL). PBLs with surface IgM expression within the lymphoid gate (PBL L IgM^+^, Figure 1A) were collected in cell culture media and used as samples for surface protein isolation by biotinylation enrichment method.

**Figure 2:**
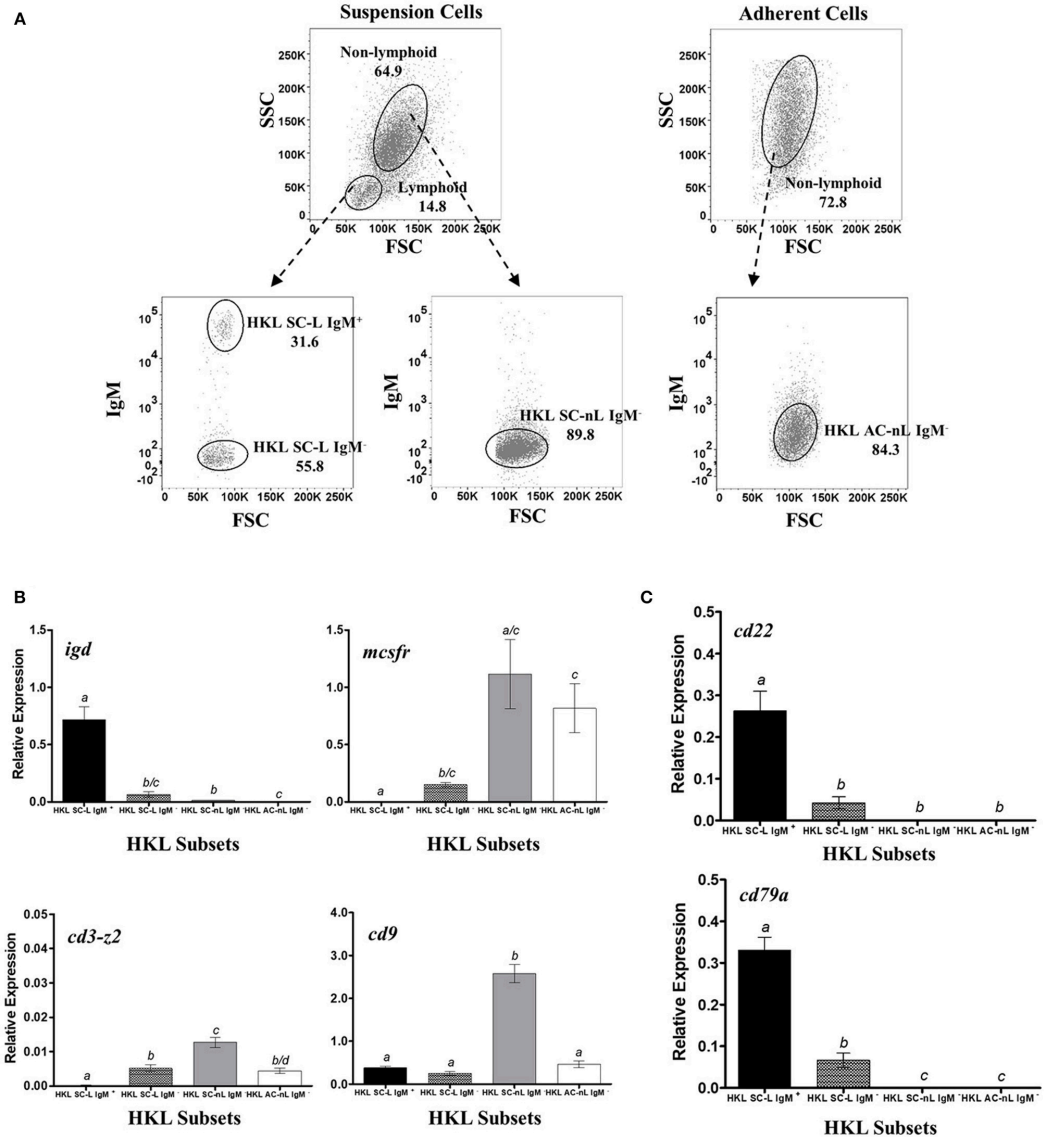
Restrictive gene expression of candidate pan-B cell markers in leukocytes from head kidney, a primary lymphoid organ of salmon. Suspension and adherent head kidney leukocytes (HKLs) were collected separately after 72 h in culture and subsequently sorted by flow cytometry based on cell size and granularity (FSC vs. SSC) and then surface IgM expression. Representative dot plot of the HKL subpopulations are shown in **(A)** with mean percentage of each fraction indicated in the graph. To ensure purity and quality of the sorted HKL subpopulations, expression of several marker genes: *igd* (B cell subset), *mcsfr* (macrophage), *cd3-z2* (T cells), and *cd9* (broad expression) were examined by RT-qPCR assay **(B)**. Upon validation of sorting protocol, gene expression of *cd22* and *cd79a* genes were subsequently determined **(C)**. Each bar represents mean relative expression data from 3 to 4 fish ± SEM. Means with different letters are significantly different (two-tailed *t*-test with Welch's correction, *p* < 0.05).

**Figure 3:**
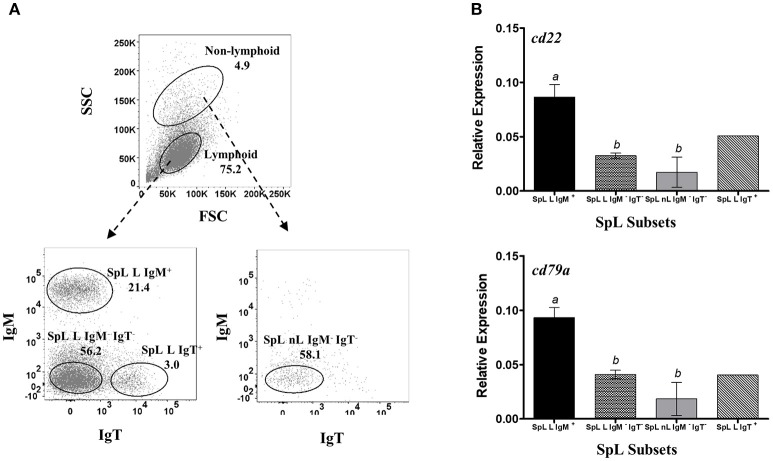
Restrictive gene expression of candidate pan-B cell markers in leukocytes from spleen, a secondary lymphoid organ of salmon. Freshly isolated splenocytes were sorted based on size and granularity (FSC vs. SSC) and then surface expression of IgM or IgT. Representative dot plot of the SpL subpopulations are shown in **(A)** with mean percentage of each fraction indicated in the graph. Cells within the lymphoid gate were sorted into IgM^+^, IgT^+^, or IgM^−^ IgT^−^ subsets (SpL L IgM^+^, SpL L IgT^+^, and SpL L IgM^−^ IgT^−^, respectively). IgM^−^ IgT^−^ cells outside the lymphocyte gate (SpL nL IgM^−^ IgT) was also collected. Expression of *cd22* and *cd79a* genes were subsequently determined in the different splenocyte subpopulations **(B)**. Each bar represents mean relative expression data from three pooled samples (five fish per pooled sample) ± SEM. Means with different letters are significantly different (two-tailed *t*-test with Welch's correction, *p* < 0.05). Due to very low cell frequency, sorted IgT data was obtained from a pool of 15 fish.

**Figure 4:**
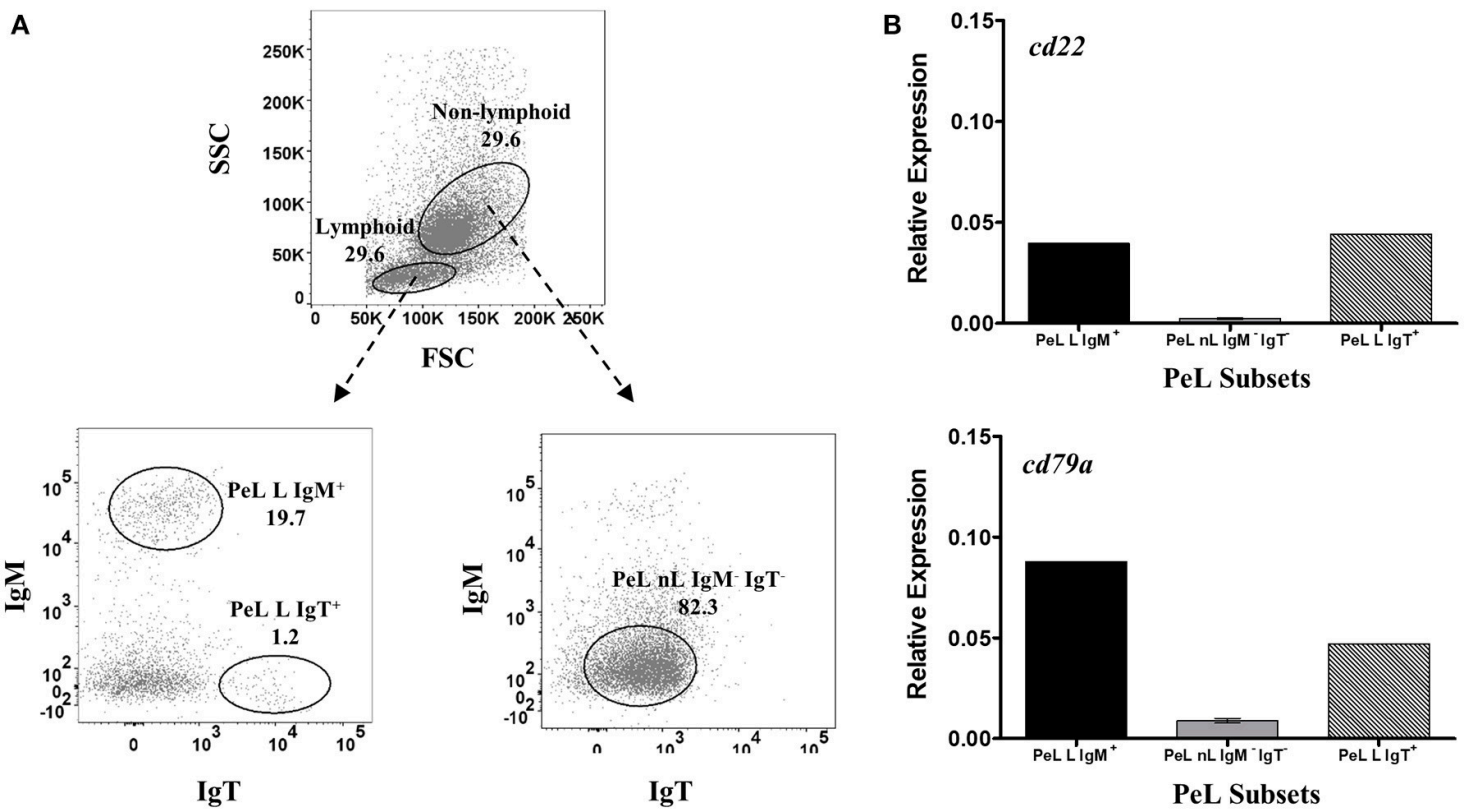
Restrictive gene expression of candidate pan-B cell markers in peritoneal cavity leukocytes of salmon. Freshly isolated peritoneal leukocytes were sorted based on size and granularity (FSC vs. SSC) and then surface expression of IgM or IgT. Representative dot plot of the PeL subpopulations are shown in **(A)** with mean percentage of each fraction indicated in the graph. PeL subsets included IgM^+^ or IgT^+^ cells within the lymphoid gate (PeL L IgM ^+^ and PeL L IgT^+^, respectively) and IgM^−^ IgT^−^ cells within the non-lymphoid gate (PeL nL IgM^−^ IgT^−^). Expression of *cd22* and *cd79a* genes were investigated in peritoneal leukocyte subpopulations **(B)**. Due to low cell frequency, PeL L IgM^+^ and PeL L IgT^+^ bars represent the relative expression data from pooled samples of 15 fish. Relative expression data for PeL nL IgM^−^ IgT^−^ was obtained from the mean of 3 pooled samples (5 fish per pooled sample) ± SEM.

For the validation of B-cell restrictive expression of candidate pan-B cell markers at the mRNA level, sorted HKL, SpL, and PeL subpopulations were used for RT-qPCR assays. Leukocytes were collected in culture either as suspension (SC) and adherent (AC) cells and stained separately as described above, and then sorted by FACS based on their FSC/SSC gating profile: lymphoid (L) vs. non-lymphoid (cells outside the lymphoid gating, nL) and their IgM and/or IgT surface expression (IgM^+^ vs. IgM^−^, IgT^+^ vs. IgT^−^). For HKLs, 4 subpopulations were obtained: SC within the lymphoid gate that was either IgM^+^ (HKL SC-L IgM^+^) or IgM^−^ (HKL SC-L IgM^−^); non-lymphoid SC that was IgM^−^ (HKL SC-nL IgM^−^); and non-lymphoid AC that was IgM^−^ (HKL AC-nL IgM^−^) (Figure 2A). SpLs, which were mostly suspension cells, were sorted as lymphoid cells with either IgM^+^ (SpL L IgM^+^); IgT^+^ (SpL L IgT^+^), or IgM^−^IgT^−^ (SpL L IgM^−^IgT^−^) surface expression; and non-lymphoid cells without IgM and IgT surface expression (SpL nL IgM^−^IgT^−^) (Figure 3A). Finally, suspension cells from PeLs were sorted as lymphoid cells expressing either IgM (PeL L IgM^+^) or IgT (PeL L IgT^+^) on their surface; and non-lymphoid cells without IgM and IgT surface expression (PeL nL IgM^−^IgT^−^) (Figure 4A). These HKL, SpL, and PeL subsets were sorted directly on RNAProtect Cell Reagent (Qiagen) and stored at −80°C until RNA extraction.

### Cell Surface Protein Isolation

Cell surface proteins from Atlantic salmon cell lines and IgM^+^ PBLs were isolated using the Pierce® Cell Surface Protein Isolation Kit (Thermo Scientific) according to the manufacturer's protocol. ASK and SSP-9 monolayers (~4 × 10^7^ cells) were quickly washed twice with ice-cold phosphate buffered saline (PBS) followed by incubation with 0.25 mg/mL Sulfo-NHS-SS-Biotin in ice-cold PBS (10 mL biotin solution per flask) on a rocking platform (100 rpm) for 30 min at 4°C. The biotinylation reaction was quenched by adding 500 μL of the provided Quenching Solution. Cells were harvested by gentle scraping, pooled, rinsed with Tris Buffered Saline (TBS), and lysed using the provided Lysis Buffer with Protease Inhibitor Cocktail (Halt™ ThermoFisher Scientific). Cells were sonicated on ice at low power using five 1 s bursts and then incubated for 30 min on ice with intermittent vortexing. The lysates were centrifuged at 10,000 × *g* for 2 min at 4°C to remove cell remnants and the resulting clarified supernatant was added to 500 μL of NeutrAvidin Agarose slurry. Biotinylated proteins were allowed to bind to the NeutrAvidin by incubating for 1 h at room temperature (RT) in the closed column with end-over-end mixing on an orbital rotator. Unbound proteins were removed by centrifugation of the column at 1,000 × *g* for 1 min followed by repetitive washing using the provided Wash Buffer with protease inhibitor. Finally, the captured surface proteins were eluted from the biotin-NeutrAvidin Agarose by incubation with 400 μL SDS-PAGE Sample Buffer (62.5 mM Tris-HCl pH 6.8, 1% SDS, 10% glycerol) containing 50 mM dithiothreitol (DTT) for 1 h at RT on an orbital rotator. The eluted proteins, representing the cell surface proteins, were collected by column centrifugation at 1,000 × *g* for 2 min.

For surface protein isolation in FACS-sorted IgM^+^ PBLs, cell suspension was centrifuged at 500 × g for 5 min at 4°C, washed twice with 3 mL ice-cold PBS to remove residual FBS, and then resuspended in 0.5 mL ice-cold PBS prior to biotinylation (1 × 10^6^ IgM^+^ cells/mL biotin solution) as described above.

To maximize the quantity of surface proteins for subsequent mass-spectrometry analysis, eluted proteins were concentrated using the Pierce™ Protein Concentrator PES 3K MWCO (ThermoFisher Scientific) following the manufacturer's protocol.

### Mass Spectrometry Analyses

Surface proteins isolated from the ASK and SSP-9 cell lines, naïve IgM^+^ PBLs, and control vs. LPS-stimulated IgM^+^ PBLs were subjected to proteomics analyses. Concentrated surface protein samples were directly lysed in 1 × NuPAGE LDS sample buffer (ThermoFisher Scientific), heated at 70°C for 10 min, and then fractionated by SDS-PAGE for 5 min at 200 V followed by Coomassie blue staining (SimplyBlue SafeStain; Thermo Fisher Scientific). Protein bands were cut and subjected to in-gel trypsin digestion before analysis by liquid chromatography-tandem mass spectrometry (LC-MS/MS).

Gel slices were subjected to in-gel reduction, alkylation, and protease digestion with 6 ng/μl trypsin (V5111; Promega) (33). OMIX C18 tips (Varian, Inc., Palo Alto, CA, USA) were used for sample cleanup and concentration. Peptide mixtures containing 0.1% formic acid were loaded onto a Thermo Fisher Scientific EASY-nLC 1,000 system and EASY-spray column (C18; 2 μm; 100 Å; 50 μm; 50 cm). Peptides were fractionated using a 2–100% acetonitrile gradient in 0.1% formic acid over 50 min at a flow rate of 250 nl/min. The separated peptides were analyzed using a ThermoFisher Scientific Q-Exactive mass spectrometer.

Raw files from the QExactive were analyzed using the Proteome Discoverer 2.2 software (Thermo Fisher). The fragmentation spectra was searched against the NCBI non-redundant (nr) *Salmo salar* 2017_1 database using an in-house Mascot server (Matrix Sciences, UK). Peptide mass tolerances used in the search were 10 ppm, and fragment mass tolerance was 0.02 Da. Peptide ions were filtered using a false discovery rate (FDR) set to 5 % for peptide identifications.

Relative protein quantitations were done using precursor ion intensities in Proteome Discoverer 2.2. To determine the relative protein amount in each sample, Exponentially Modified Protein Abundance Index (emPAI) values were extracted from mascot search results.

### RNA Isolation and cDNA Synthesis

Total RNA from sorted cells was extracted using either the RNeasy® Mini (≥500,000 cells) or Micro (< 500,000 cells) Kits (Qiagen), with in-column DNAse I treatment (Qiagen) according to the manufacturer's protocol. For sorted HKL, SpL, and PeL subsets, an extra centrifugation step at 5,000 × g for 10 min was performed to remove the RNAProtect Cell Reagent before proceeding to RNA extraction of the cell pellet. RNA yield and purity were determined using Nanodrop ND-1,000 Spectrophotometer (Nanodrop Tec. Wilmington, DA, USA) and stored at −80°C. Isolated RNA (75–150 ng) was reverse transcribed into cDNAs in 20 μl reaction volumes using the QuantiTect Reverse Transription Kit (Qiagen) following the manufacturer's protocol. Resulting cDNA was diluted 1:5 and stored at −20°C until further use.

### Gene Expression Analyses

Expression levels of RNA transcripts of selected genes were analyzed by RT-qPCR on an ABI Prism 7500 FAST Cycler (Applied Biosystems). cDNAs from sorted HKL subpopulations (2.5 ng total cDNA input) was used per qPCR reaction (20 μL final volume) using the Fast SYBR® Green Master Mix (Applied Biosystems). For SpL and PeL subsets, total cDNA input was 1.5 ng. Information for all primers used is listed in Table 1. The efficiency of the amplification was determined for each primer pair using serial 2-fold dilutions of pooled cDNA, and only primer pairs with efficiencies between 1.90 and 2.10 were used. Each sample was measured in duplicate under the following conditions: 95°C for 20 s followed by 40 cycles of 95°C for 3 s and 60°C for 30 s.

**Table 1:** Primers used for SYBR green qPCR assays.

**Genes**	**Accession no**.	**Oligo name**	**Sequence (5′-3′)**	**Product size (bp)**	**Efficiency**
***ef1αβ***	NM_001123629.1	Fwd	CCCCTCCAGGACGTTTACAAA	57	1.97
		Rev	CACACGGCCCACAGGTACA		
***igd***	XM_014203123.1^a^	Fwd	CCAGGTCCGAGTGGGATCA	136	1.92
		Rev	TGGAGCAGGGTTGCTGTTG		
***mcsfr***	NM_001171807.1	Fwd	CACCAGTAACCCTAACCACTTC	97	2.00
		Rev	GACCTGCTTGTCCTGCATTA		
***cd3-z2***	NM_001123620.1	Fwd	ATTCTGGATGGCTTCCTCCT	144	2.03
		Rev	TATTCGCCCATAACCACCTC		
***cd9***	XM_014187224.1	Fwd	GAGGCCTTGAAGGAGACATTAC	115	2.00
		Rev	CCTCCAGTCCTTCCTTCTTTG		
***cd22***	XM_014165590.1	Fwd	GCCAGAGGACAAAGGTCATTA	106	2.04
		Rev	CTGAGTGTATCTTGGAACATAGGAA		
***cd79a***	XM_014187908.1	Fwd	TCTGAACGACTCAGGGTTGTA	105	2.02
		Rev	TTCACCATCGGCCTGTAGA		

a *Used qPCR primers previously designed by Tadiso et al. (34)*.

The expression of individual genes was normalized to that of Atlantic salmon elongation factor 1αβ (EF1αβ) and presented as relative expression using the 2^−Δ*Ct*^ method, where ΔCt is determined by subtracting the EF-1α value from the target Ct as described previously (35, 36). Negative controls with no template were included in all experiments. A melting curve for each PCR was determined by reading fluorescence every degree between 60 and 95°C to ensure only a single product had been amplified.

Statistical analyses of RT-qPCR data were performed in GraphPad Prism 5.04 using a two-tailed student *t*-test with Welch's correction when the *F* test indicated that the variances of both groups differed significantly. The differences between the mean values were considered significant when *p* ≤ 0.05.

## Results

Our mass spectrometry approach was able to identify a combined dataset of 3,140 proteins from our surface protein-enriched salmon B cell and/or HK cell line samples (full list of identified proteins is available at https://doi.org/10.18710/JU3DWE), of which 21% were deemed to be membrane proteins by GO-annotation or transmembrane predictions. This percentage, however, may have been underestimated due to incomplete annotation in some Atlantic salmon proteins. We subsequently focused on surface protein orthologs that were previously reported as part of the CD molecules in mice and/or humans.

### CD Proteins Exclusively Identified From Atlantic Salmon HK Cell Lines

A total of 38 and 39 CD proteins were identified from ASK and SSP-9 cells, respectively (Figure 1B). Nine CD proteins were detected only in ASK cells, while 11 CD proteins were detected only in SSP-9 cells. Twenty-four CD proteins were shared between these two cell lines.

### CD Proteins Common to Atlantic Salmon B Cells and HK Cell Lines

In agreement with the established broad expression of CD81 in mammals (37), this multifunctional tetraspanin protein was detected in high quantities in all proteomics samples (Table 2). Similarly, ubiquitously expressed transmembrane glycoproteins (38–40), CD98 and CD147 were detected in salmon ASK, SSP-9, and IgM^+^ peripheral B cells. CD147 can bind directly to CD98, which associates with integrins, which is in turn involved in cell adhesion, fusion, proliferation, and growth (41). CD98 was more abundant in LPS-stimulated B cells than control (relative abundance > 2.00; Table 2).

**Table 2:** CD molecules identified in Atlantic salmon IgM^+^ B cell samples.

**CD molecule**	**Protein name**	**Known expression of mammalian homolog**	**Function of mammalian homolog**	**Accession no**.	**Mascot score**	**Sequence coverage**	**No. of peptides**	**No. of PSMs**	**No. of unique peptides**	**emPAI in naïve B cells^a^**	**Relative abundance (LPS/Control)**
CD9	Tetraspanin-29 (Tspan-29)	Leukocytes, epithelial and endothelial cells	Cell adhesion, migration, signal transduction	XP_014042699.1	395	29	4	12	4	0.52	1.17
CD11a	Integrin alpha-L-like (ITGAL)	Exclusive to leukocytes	Leukocyte-endothelial cell interactions, cell adhesion, differentiation/development	XP_013986946.1	292	7	6	9	6	–	1.17
CD11c	Integrin alpha-X-like (ITGAX)	Exclusive to leukocytes	Adhesion, cell migration, survival, and proliferation	XP_014014772.1	273	7	7	9	7	0.09	1.59
CD18	Integrin beta-2-like (ITGB2)	Exclusive to leukocytes	Signal transduction, adhesion	XP_014019758.1	2,358	34	20	85	20	1.33	1.19
CD22	SIGLEC-2	Exclusive to B cells	Immunoregulation, B cell adhesion, BCR co-receptor, signal transduction	XP_014021065.1	329	8	8	13	8	0.19	1.53
CD40	Tumor necrosis factor receptor superfamily member 5 (TNFRSF5)	B cells, monocytes, antigen-presenting cells, endothelial and epithelial cells	Cell adhesion, cell proliferation, and signal transduction	NP_001134708.1	64	9	3	4	3	–	8.60
CD45	Protein tyrosine phosphatase receptor type C (PTPRC)	All hematopoietic cells except erythrocytes and plasma cells; B220 isoform exclusive on murine B cells	Regulator of B- and T-cell antigen receptor signaling, cell growth and differentiation	XP_013979041.1	5,714	42	43	207	43	1.79	1.66
CD53	Tetraspanin-25 (Tspan-25)	Exclusive to hematopoietic cells	Cell adhesion, activation, and migration	NP_001134048.1	51	3	1	2	1	–	1.40
CD63	Tetraspanin-30 (Tspan-30)	Leukocytes, endothelial cells	Complexes with integrins, regulation of cell growth and motility	XP_013991669.1	192	9	2	8	2	–	0.98
CD68	Macrosialin	Hematopoietic and non-hematopoietic cell types	Phagocytosis, macrophage homing	NP_001158857.1	34	3	1	1	1	–	0.79
CD79A	B cell antigen receptor complex-associated protein alpha chain; MB-1 membrane glycoprotein	Almost exclusive to B cells	Part of the BCR complex, required in B cell signaling	XP_014041898.1 XP_014010819.1	84 72	12 13	3 3	4 5	1 1	0.30 0.13	1.71 1.22
CD81	Target of the Antiproliferative Antibody 1 (TAPA-1); Tetraspanin-28 (Tspan-28)	Broad expression	B cell activation, cell adhesion, stimulation, differentiation/development	XP_014031360.1	1,139	28	4	31	1	0.30	1.43
CD84-H1	Signaling lymphocyte activation molecule 9 (SLAM-9)	Macrophages, monocytes, lymphocytes	Long-term humoral immune response	XP_014062779.1	50	3	1	2	1	0.09	0.01^b^
CD87	Urokinase plasminogen activator surface receptor-like	Monocytes and granulocytes, T lymphocytes, NK cells	Cell adhesion, migration, chemotaxis, proliferation, receptor/co-receptor	XP_014012019.1	1,255	54	7	32	5	1.01	1.10
CD98	4F2 cell-surface antigen heavy chain-like	Lymphocytes, NK cells, macrophages, granulocytes, endothelial and epithelial cells	Activation/costimulation, immunoregulation	XP_013984627.1 XP_014051944.1	1,664 1,397	40 40	12 13	44 30	10 11	– –	3.93 2.28
CD147	Basigin	Hematopoietic cells, epithelial and endothelial cells	Regulation of immune responses, lymphocyte activation, adhesion, cell recruitment	XP_014071798.1	296	19	4	10	4	–	1.36
CD156c	A disintegrin and metalloproteinase domain-containing protein 10 (ADAM-10)-like	Broad expression	“Molecular scissors” important in leukocyte regulation	XP_014025741.1	52	4	2	2	2	–	N/A^c^
CD184	C-X-C chemokine receptor type 4-like (CXCR4)	Broad expression	Receptor/coreceptor, chemotaxis	NP_001158765.1	121	2	1	4	1	–^d^	1.53
CD185	C-X-C chemokine receptor type 5-like (CXCR5)	Broad expression	Homing and cell movement and migration	XP_014071056.1	70	3	1	1	1	0.10	1.24
CDw199	C-C chemokine receptor type 9-like (CCR9)	Leukocytes	Important in B cell migration, maturation, and function	XP_014043995.1	78	3	2	4	2	–	1.15
CD282	Toll-like receptor 2 (TLR2)	Monocytes, granulocytes, B cells, resting T cells,	Recognition of bacterial lipopeptides and subsequent activation of innate immune responses	XP_014061839.1	29	2	1	1	1	–	0.88

*PSM, peptide-spectrum match; emPAI, exponentially modified protein abundance index*.

a *No emPAI value for proteins with weak positive hits*.

b *Below detection threshold in LPS-stimulated B cell sample*.

c *Below threshold level for relative abundance ratio*.

d *Detected in 1 of 2 naïve/control-stimulated B cell samples*.

The tetraspanin CD63 and transmembrane protein CD156c, also known to be expressed in many cell types (42, 43), were identified in both ASK and B cells. CD63 functions as a transport regulator implicated in intracellular protein trafficking (44). It has been shown to down-regulate CD184 (CXCR4) by serving as a molecular target of the transcriptional repressor Bcl6 (45). CD156c (ADAM10) functions as a molecular scissor that cleaves the extracellular regions of its transmembrane target proteins, which is an important mechanism for the regulation of leukocyte development and function (43).

The transmembrane protein receptor CD40 was detected in both SSP-9 and IgM^+^ B cells. This member of the TNFR superfamily has been initially characterized on B cells and subsequently found to be expressed on antigen-presenting cells, and many other immune and non-immune cells (46). The binding of CD40 to its CD154 ligand (CD40L) regulates a wide spectrum of cellular processes, including the activation, proliferation, and differentiation of B cells (47). CD40 exhibited the highest increase in salmon B cell surface expression upon stimulation with a TI antigen, with relative abundance value of 8.60 in LPS-treated vs. control B cells (Table 2).

### CD Proteins Exclusively Identified From Atlantic Salmon B Cells

Out of the 21 total CD molecules Identified from salmon IgM^+^ PBLs, 15 were found to be present in these B cell samples only and were not detected in the two salmon hematopoietic organ-derived cell lines (Figure 1B). The receptor-like protein tyrosine phosphatase, CD45, reported to be one of the most abundant cell surface glycoproteins expressed on mammalian leukocytes (48), was the most abundant protein detected in the salmon B cell samples with a relative quantitation value of 1.79 as estimated by emPAI (Table 2) based on protein coverage by the peptide matches in the database search result (49).

#### B Cell-Restricted Proteins (CD22 and CD79A)

Two surface proteins (CD22 and CD79A) that previously have been used as B cell-exclusive markers in mammals were identified in the salmon B cell samples. CD22 is a transmembrane glycoprotein that belongs to the sialic acid-binding immunoglobulin-like lectins (SIGLEC) family that serves as a BCR co-receptor (50, 51). In mammals, its expression is predominantly exclusive to subsets of mature B cells, with surface expression appearing simultaneously with surface IgD and is subsequently lost on plasma cells (52–54). CD22 is a well-established regulator of innate and adaptive B cell responses in mammals [reviewed in (55)]. One of the main functions of CD22 is to down-regulate the activation threshold of BCR through its association with tyrosine phosphatases and other signaling molecules (51, 56). In addition to BCR signaling, several initial studies in mice have shown that CD22 also regulates TLR signaling and the survival of B cells (55). However, it has been suggested that CD22 likely functions differently between innate-like B-1 and conventional B-2 cells since CD22 is differentially regulated after BCR-mediated and -independent activation in these B cell lineages (57).

CD79A, on the other hand, is an integral membrane protein belonging to the Ig gene superfamily that associates with membrane Ig on B cell surface and, together with CD79B, forms the signal transduction region of the B cell antigen receptor (BCR) complex (58, 59). Due to their importance in B cell development and induction of B cell activation, CD79 proteins are expressed in virtually all subsets of B cells (60), from the very early stages of B cell development to plasma cells (61–65).

#### Tetraspanins (CD9 and CD53)

Of the four tetraspanin proteins that were identified by proteomics, detectable quantities of CD9 and CD53 were only present in salmon B cells, despite the reported surface expression in different hematopoietic cell types (66, 67) and/or endothelial cells (68) of their mammalian homologs. This protein family consists of four-span transmembrane proteins that have been described as “master organizers” of the plasma membrane (69) and “molecular facilitators” (42) in a variety of biological processes.

#### β_2_ Integrins (CD11a, CD11c, and CD18)

CD18 (ITGB2), CD11a (ITGAL), and CD11c (ITGAX) that were detected on salmon B cell surface belong to the β_2_ integrin family of adhesion and signaling molecules. Integrin β chain subunit CD18 can pair with one of four alpha chain subunits (CD11a, CD11b, CD11c, or CD11d) to form the CD11/CD18 complex that play important roles in the recruitment of immune cells to sites of inflammation, cell–cell contact formation, and regulation of downstream effects on cellular signaling (70). In mammals, these β_2_ integrin complexes are found exclusively on leukocytes, particularly myeloid cells and NK cells, and to a lower expression level, B and T lymphocytes.

#### Chemokine Receptors: CD184, CD185, and CDw199

Three chemokine receptors, CD184 (CXCR4), CD185 (CXCR5), and CDw199 (CCR9) were identified in Atlantic salmon B cells. These are G-protein coupled, seven-transmembrane receptors with CD184 and CD185 classified into the C-X-C (alpha) class and CDw199 into the CC (beta) class. CD184 and CD185 are known to have broad expression across many cell types, including lymphocytes, endothelial, epithelial and hematopoietic stem cells (71). Similarly, CDw199 is expressed in different leukocyte subpopulations such as macrophages, dendritic cells, T cells, and B cells (72–75). These chemokine receptors are important in the migration, maturation, and function of B cells (76–78).

#### Other Surface Proteins (CD68, CD84-H1, CD87, and CD282)

In addition to members of the protein families described above, several other surface proteins were found present on salmon B cells. These included CD68, CD84-H1, CD87, and CD282.

CD68, the human homolog of macrosialin, is a highly glycosylated type I transmembrane protein belonging to the lysosomal-associated membrane protein (LAMP) family of glycoproteins (79). While this protein was initially regarded as a macrophage marker (80), CD68 expression on other hematopoietic and non-hematopoietic cell types have been subsequently reported, including B cell lines (81–83).

CD84-H1 (a.k.a. CD2-F10 and SLAM9) belongs to the signaling lymphocyte activation molecule (SLAM) family of cell surface receptors within the immunoglobulin superfamily (84, 85). This protein has been shown to be widely expressed in many immune cells of humans, including B cells (84, 85). However, the exact function of CD84-H1 in B cells is still unclear. In general, SLAM proteins are said to contribute to the generation of long-term humoral immune response (86, 87).

CD87, also called urokinase plasminogen activator receptor (uPAR), is a surface glycoprotein that mediates a wide range of biological processes beyond plasminogen activation, including cellular adhesion, migration, chemotaxis, and proliferation [reviewed in (88, 89)]. In humans, CD87 is known to be highly expressed on monocytes and granulocytes, particularly on mature cells (90). As such, it is used as a surface marker for terminal granulocytic maturation (91). Resting B and T lymphocytes and cell lines of lymphoid lineage do not seem to express CD87 (90, 92), although surface expression has been reported on activated T cells and NK cells (90). Next to CD45 and CD18, salmon CD87 had the highest emPAI obtained in naïve B cells, with relative abundance comparable between control and LPS-activated B cell samples.

Another surface protein detected on salmon B cell surface was CD282, more commonly known as toll-like receptor 2 (TLR2), a membrane-bound protein that recognizes the evolutionarily conserved bacterial lipopeptides (93). In humans, surface expression of CD282 is found to be highest on innate cells such as monocytes and granulocytes (94). Detectable level of CD282 expression has also been observed on activated but not resting T cells (95, 96), as well as on surface of different B cell subpopulations, albeit mostly at low level (94, 97, 98).

### B Cell-Restrictive Gene Expression of Candidate Salmon Pan-B Cell Markers

Given the known B cell-exclusive surface expression of their mammalian homologs, CD22 and CD79A were shortlisted as potential pan-B cell markers for salmon. In the absence of definitive Abs against these candidate markers, we resorted to qPCR assays for validating their B cell restrictive expression using leukocyte subsets from different lymphoid organs.

Sorted cells from HK (Figure 2A) were used as the main source of different leukocyte subpopulations for the RT-qPCR validation assays. As the major hematopoietic organ in teleost, HK consists of a heterogeneous mixture of leukocytes belonging to the lymphoid and myeloid lineages (99). HKL subsets were first examined for gene expression of known markers for B cells, macrophages, and T cells in order to confirm the nature of cells present in each subset, as well as to ensure that no cross-contamination of cell subsets occurred during FACS sorting.

As expected, only the lymphoid subsets had detectable *igd* expression, with transcript levels highest in the HK suspension cell IgM^+^ lymphoid subset (HKL SC-L IgM^+^, Figure 2B), in accordance with the reported dual IgM and IgD expression in majority of naïve mature peripheral B cells of mammals [reviewed in (100–102)] and trout (103). Significant albeit much lower *igd* expression was also observed in HK suspension cell IgM^−^ lymphoid subset (HKL SC-L IgM^−^), which could suggest the presence of IgD^+^ only B cells in naïve HKLs. By contrast, the suspension and adherent IgM^−^ non-lymphoid subsets (HKL SC-nL IgM^−^ and HKL AC-nL IgM^−^, respectively), assumed to consist of myeloid cells (macrophages, monocytes, and/or granulocytes), had very low to non-existent *igd* expression.

The HK suspension cell IgM^+^ lymphoid subset had no detectable transcription of the macrophage marker, *mcsfr*, which was expressed in high levels in the putative myeloid cell subsets, HKL SC-nL IgM^−^ and HKL AC-nL IgM^−^. Detectable levels of *mcsfr* transcripts was present in the HK suspension cell IgM^−^ lymphoid subset, which could indicate the presence of contaminating macrophages in this leukocyte subpopulation. Similarly, no detectable *cd3-z2* gene expression was observed in the HK suspension cell IgM^+^ lymphoid subset, while the remaining HKL subsets exhibited relatively low expression of this T cell marker.

Additionally, to ensure that any observed absence of detectable transcripts was not due to poor cDNA/RNA quality of our samples, the mRNA levels of the broadly expressed CD9 tetraspanin were also determined in the sorted HKL subpopulations. In general, relative expression of *cd9* was comparably high across all HKL subsets, with the HK suspension cell IgM^−^ non-lymphoid subset having the most expression.

The gene expression of our candidate B cell markers was subsequently investigated. HK suspension cell IgM^+^ lymphoid subset with a putative B cell phenotype (i.e., significant gene expression of *igd* and *cd9*, but not *mcsfr* and *cd3-z2*) exhibited the highest *cd22* and *cd79a* expression (Figure 2C). By contrast, very low to non-existent mRNA levels were observed in both the suspension and adherent IgM^−^ non-lymphoid subsets (HKL SnL IgM^−^ and HKL AnLIgM^−^, respectively), consisting mostly of cells from the myeloid lineage and some T lymphocytes as per *mcsfr* and *cd3z* gene expression profile. Low but detectable levels of *cd22* and *cd79a* were also observed in the HKL SL IgM^−^ subset, likely due to the presence of IgD^+^ and IgT^+^ B cells in this leukocyte subpopulation.

To check whether the apparent B cell restrictive expression of *cd22* and *cd79a* is consistent across different B cells from different lymphoid sources, we also performed the same gene expression assay in leukocyte subsets from spleen (Figure 3A) and peritoneal cavity (Figure 4A). IgT subpopulation was also added in the analysis to determine *cd22* and *cd79a* expression in a non-IgM B cell isotype. Similar to what was observed in HKLs, highest and lowest *cd22* and *cd79a* mRNA levels were obtained in the spleen IgM^+^ lymphoid (SpL L IgM^+^) and IgM^−^ non-lymphoid subsets (SpL nL IgM^−^), respectively (Figure 3B). High mRNA expression for these candidate markers was also found in the spleen IgT^+^ lymphoid subset (SpL L IgT^+^) as well as in the IgM^−^ IgT^−^ lymphoid subset (SpL L IgM^−^ IgT^−^), which could contain IgD only B cells.

For PeLs, comparable levels of *cd22* transcription were observed between IgM^+^ (PeL L IgM^+^) and IgT^+^ (PeL L IgT^+)^ lymphoid subsets, while very low *cd22* gene expression was obtained in the PeL IgM^−^ IgT^−^ non-lymphoid subset (PeL nL IgM^−^ IgT^−^) (Figure 4B). Gene expression of *cd79a*, on the other hand, was higher in the IgM^+^ than IgT^+^ peritoneal lymphoid cells. Lowest *cd79a* expression was observed in the IgM^−^ IgT^−^ non-lymphoid subset of the peritoneal leukocytes (PeL nL IgM^−^ IgT^−^).

## Discussion

To our knowledge, this is the first report on the profiling of salmon B cell surface protein repertoire. Although proteomics has been employed in some investigations of fish immune responses to various infections [reviewed in (104)], it is not commonly used to characterize the surface protein expression of specific immune-related cells in teleosts. A previous proteomics study on Atlantic salmon focused on profiling changes in fish serum proteins following infection with salmonid alphavirus (105). Our approach focused on B cells and was useful in the identification of 21 CD molecules from salmon IgM^+^ B cells. The relatively limited number of CD proteins identified could be partially explained by the initial low number of B cells in the starting material (3 × 10^6^ viable IgM^+^ B cells after 72 h in culture) used in the surface protein isolation. In addition, despite being one of the popular choices for cell surface proteome profiling, the biotinylation technique employed for the enrichment of plasma membrane proteins may not be the most effective method for isolating glycosylated proteins (106). Since majority of cell surface proteins are glycosylated (107), many surface glycoproteins may not have been included in our mass spectrometry samples.

Peripheral blood was used as source of salmon B cells for proteomics due to higher total leukocyte yield [i.e., 15 × and 4 × more total leukocytes than the spleen and the HK, respectively (108)] and the abundance of IgM^+^ B cells in this compartment (22). For comparison, surface proteins from two Atlantic salmon head kidney cell lines, ASK and SSP-9, were also identified. Although the exact cell composition of these hematopoietic-tissue derived cell lines is unknown, these seem to include cells from the myeloid lineage based on their respective proteomics profile. Given its epithelial-like morphology and previously reported gene expression profile (31), SSP-9 is likely comprised of macrophage-like cells. Specifically, majority of the identified SSP-9-exclusive surface proteins (CD2, CD34, CD74, CD151, CD205) are associated with expression on a macrophage subset with antigen-presenting capacity (109–113). Detection of CD203c, a known basophil and mast cell marker in humans (114), suggest that SSP-9 may contain granulocytes.

### Salmon IgM^+^ B Cell Surface Proteome

All the surface proteins identified from our salmon B cell samples have been previously detected on the surface of mammalian B cells, except for CD87. In teleost, only a few of these B cell-associated proteins have been studied so far. Constitutive gene expression of tetraspanins *cd9* and *cd63* have been reported in sorted IgM^+^ B cells of trout from different immune organs (115). Surface expression of CD22 has also been shown in PBLs of tongue sole (116).

In keeping with their established roles in B cell activation, CD40 (117) and CD98 (118) surface expression were significantly higher in LPS-stimulated IgM^+^ B cell samples. Co-expression of CD40 and IgM^+^ has been established on B cell surface of zebrafish, with significant up-regulation of CD40 similarly observed following LPS treatment (119). In addition, modest increase in CD40 transcripts has been reported for Atlantic salmon IgM^+^ B cells in the presence of another TLR agonist, CpG oligodeoxynucleotides (22).

Surprisingly, CD87, which has been reported to be absent on surface of resting B lymphocytes and B cell lines of mammals (90, 92), was detected in our naïve salmon IgM^+^ B cell samples. It should be noted that CD87 acts as a ligand for β_2_ integrins such as CD18 and CD11a/c identified on salmon B cells, and thus usually found in close association with these protein complexes on leukocytes (120). CD87 has been demonstrated to mediate cell-cell adhesion by interaction with integrins on the same as well as apposing cells (121). Hence, the CD87 proteins detected in our proteomics experiment may be an artifact from a CD87-β_2_ integrin complex formed by trans-interaction of salmon B cells with CD87-expressing monocytes or granulocytes. It is unlikely that the proteins were contributed directly by contaminating monocytes or granulocytes since the samples were checked for purity (>98% IgM^+^ PBLs) before surface protein isolation. Alternatively, it is also possible that salmon, in contrast to mammals, express CD87 on their B cell surface.

Peripheral blood provides the means for cells to move systemically within the animal body; hence, one would expect a mixture of circulating B cell subsets in this compartment. Surface detection of chemokine receptors CXCR4, CXCR5, and CCR9 important in trafficking naïve B lymphocytes (76) seem to suggest that our peripheral IgM B cell population is comprised of a migrating population of B cells from different lymphoid compartments. In humans, the majority of peripheral B cells are of the conventional B-2 cell type bearing both membrane IgM and membrane IgD, which accounts for about 10% of total PBLs (122). Given the current technical constraints, it was not possible to further sort the largely undefined subsets of the salmon IgM^+^ peripheral B cell subpopulation.

More recently, it has been shown that, contrary to the dogma that all plasma cells have permanently switched off expression of membrane-bound Ig molecules to produce their secreted version (antibodies), some mature plasma cells in mice retain their expression of surface IgM and functional BCR (123, 124). Thus, it is possible that our LPS-treated peripheral B cells contained some differentiating IgM^+^ plasmablasts, plasma cells, or memory B cells. However, none of the known markers for these differentiated B cells [i.e., CD27 for memory B cells (125), CD138 for plasma cells (126)] was identified in the stimulated B cell samples. Based on previous studies in trout, this can be partially explained by the relatively short stimulation period (72 h incubation in culture) used in our study. In naïve trout PBLs, significant antibody secreting cell response is observed only after 4 days of LPS stimulation using 4 times higher dose, with peak responses occurring by Day 7 in culture (127). In addition, memory B cell responses to TI and TD antigens are observed only after 4–7 days of *in vitro* re-stimulation of PBLs from previously immunized trout (108).

It should be emphasized that several other mammalian pan-B cell and subset markers known to be encoded in the Atlantic salmon genome were not detected in our salmon IgM^+^ B cell samples. Failure to detect a particular surface protein should be interpreted with caution due to previously indicated technical limitations of our experimental approach or their presence at low frequency in our heterogeneous pool of IgM^+^ B cell subpopulations. Moreover, it should be noted that our shortlist of salmon CD proteins was based on their mammalian counterparts. Other surface proteins that were detected exclusively in the IgM^+^ samples but without homologs or are not classified as CD molecules in mammals were not included in this list. Further investigation of the “non-CD” surface proteins is therefore needed in order to determine whether or not these can serve as unique B cell markers in salmon.

### Identification of CD22 and CD79A as Potential Salmon B Cell Markers

Due to lack of access to antibodies against salmon IgD and myeloid markers, our analysis was constrained to leukocyte subpopulations sorted by cell size and granularity and subsequent positive selection for IgM^+^ or IgT^+^ cells, representing independent salmon B cell lineages. Despite this clear limitation, our current sorting protocol is still a valid useful tool in studying the different salmon leukocyte populations as evidenced by the results of our qPCR validation assays using different leukocyte markers.

In general, *cd22* vs. *cd79a* gene expression patterns of the different leukocyte subsets were comparable across different lymphoid organs. Highest relative expression were observed in all IgM^+^ B cell subsets, while very low to below detectable level of expression were found in IgM^−^/IgT^−^ non-lymphoid subset (likely consisting of myeloid cells). Relatively lower but significant level of expression was also found in non-IgM subsets within the lymphoid gate, which likely consist of either a mixed population of IgT^+^ or IgD^+^ only B cells (i.e., HKL SC-L IgM^−^) or IgD^+^ only B cells (i.e., SpL L IgM^−^ IgT^−^). While generally regarded as B cell-exclusive proteins (52, 128), it is important to note that mammalian CD22 and CD79A have also been reported to be expressed on the surface of some non-B cell populations, including T cells (129) and various myeloid cells (130–133). However, this is likely not the case for Atlantic salmon given the negligible transcript levels of *cd22* and *cd79a* in all the myeloid-containing leukocyte subsets tested.

Although mRNA levels do not necessarily correlate directly with surface protein expression, gene expression profiles of salmon *cd22* and *cd79a* clearly showed that the B cell exclusivity of our candidate CD molecules extend down to the level of transcription. This B cell-restrictive expression was consistent across different lymphoid compartments (peripheral blood, head kidney, spleen, peritoneal cavity) and different Ig isotypes (IgM and IgT), thus providing strong support to the potential of CD22 and CD79A as pan-B cell markers for salmon.

Extensive gene duplications in the Atlantic salmon genome (134, 135) should be taken into account in finding appropriate B cell markers. Specifically, Atlantic salmon encodes an unusually high number of CD22-like paralogs and isoforms (Supplementary Table 1) that could have different regulatory expression and conformation on B cells. Interestingly, peptide sequences specific to only one CD22 paralog was detected on the salmon B cell surface in our mass spectrometry analysis. Based on our qPCR assay, mRNA expression of this particular variant was also specific in salmon B cells. In humans, a CD22 molecule with a different conformation to that found on B cells has been detected on basophils (130). Whether other CD22 variants/isoforms are also expressed in other subpopulations of salmon leukocytes, or in specific subsets of salmon B cells, is still unknown, but is beyond the scope of this study. For simplicity, the particular CD22 paralog (XP_014021065.1) identified in this study was referred as CD22.

### Other Potential B Cell Markers

CD45, the most abundant surface protein identified in our salmon B cell samples, exists in different alternative splicing isoforms, which are expressed in cell-type specific patterns on functional subsets of mammalian lymphocytes (48). In mice, a long isoform of CD45 called B220 (136) has been used as a pan B-cell marker due to its specific expression throughout stages of B cell development, including entry into the memory B cell pool (137). A similar long CD45 isoform (CD45RABC) has been identified in human B cells (96), which has been used as marker for certain human B cell subsets (138). This B-cell specific CD45 isoform in mammals consists of all 33 exons of the CD45 gene (139). Atlantic salmon genome encodes for seven CD45 transcript variants in Chromosome 10 (Supplementary Table 1), with alternate splicing of three exons (4, 5, 6) producing six CD45 isoforms [GenBank Assembly Accession No. GCA_000233375.4 (135)]. Given that all exons are present in the B cell-specific isoform, it was not possible to determine whether the long CD45 isoform was the only isoform detected on salmon B cell surface. Hence, further investigation for the presence of other CD45 isoforms in sorted salmon B cell subpopulations (i.e., qPCR assays using primers specific for the other splice variants) is still needed before its potential as another salmon pan-B cell marker can be fully appreciated.

Several other CD molecules identified on salmon B cell samples can be explored for their potential in discriminating specific B cell subsets despite their non-exclusive B cell surface expression. Murine CD9 is expressed on the surface of innate-like B cells and plasma cells, but not on naïve conventional B2 cells (140, 141). In our proteomics data, CD9 was detected in high abundance in all samples of salmon IgM^+^ peripheral B cells but not in either of the two salmon head kidney cell lines. This implies that the salmon IgM^+^ peripheral B cell subpopulation are comprised of cells with significantly high expression of this protein. In rainbow trout, *cd9* mRNA levels are similar in IgM^+^ and IgM^−^ B cells from blood and HK (115). In agreement with this, comparable levels of *cd9* expression was also observed in the IgM^+^ and IgM^−^ fractions of our sorted HKL lymphoid cells, but highest constitutive gene expression was still found in the myeloid fractions. Thus, while CD9 cannot be used as the sole and primary marker for salmon B cells, its potential for identifying specific B cell subsets should be explored further.

Other potential B cell subset markers are the CD11 β_2_ integrins. In contrast to mammals wherein four β_2_ integrins have been identified, Atlantic salmon genome encodes only two: CD11a and CD11c. Both of these proteins were detected in our salmon IgM^+^ B cell samples, which could suggest their potential use as markers to functionally-equivalent B cell subsets in mammals. High surface CD11a expression has been associated with a new subpopulation of IFN-γ-secreting innate B cells (142). Expression of CD11c has also been recently used as marker for distinct B cell subsets in mice and humans (143, 144), including CD11c^low^-expressing plasmablasts that predominantly secrete Ag-specific IgM antibodies in a T cell-independent manner (145) and CD11c^+^ ‘atypical memory B cells’ (144).

Interestingly, CD11b, which is used as a marker to differentiate B1 cells that reside in the peritoneal cavity and those that recently migrated into the spleen (146), has not been detected until present. Among salmonids, only coho and Chinook salmon have CD11b-like protein recorded in the sequence database. However, the salmonid sequences of CD11c (ITGAX) is very similar to that of CD11b (70). In fact, Atlantic salmon CD11c (XP_014014772.1) has 70 and 90% homology with CD11c-like proteins of coho (XP_020308950.1) and Chinook (XP_024269535.1) salmon, respectively. Similarly, CD11d, which is only found in coho (XP_020334852.1), has 95% homology with Atlantic salmon CD11a (XP_014032228.1). At this point, it is unknown whether Atlantic salmon CD11c-like and CD11a-like proteins have overlapping functions with the mammalian CD11b and CD11d proteins, and whether expression patterns of these mammalian B cell subset markers are similar to their ancestral counterpart in teleosts.

### Salmon IgM^+^ B Cell Surface Phenotype Is Consistent With Innate-Like B Cells of Mammals

It is worthy to note that several of the surface proteins (CD9, CD11a, CD11c) identified on salmon IgM^+^ B cells in high abundance are associated with innate-like B cells in mice and humans. CD22, one of the identified potential salmon B cell marker in this study, is also known to be expressed the highest in MZ B cell precursors out of any mammalian B cell subset (147). Additionally, detection of CD282 (TLR2), which has been previously shown to be expressed higher in naïve innate-like B cells than conventional B2 cells (97), implies a possible function of salmon IgM^+^ peripheral B cells in TI-responses. Altogether, these are consistent with a salmon IgM^+^ B cell phenotype closer to innate-like B cells (B1 and MZ B2) than conventional B2 cells in mammals. This supports the previously proposed B cell evolution hypothesis (18) which suggests that teleost IgM B cells are the ancestors of the innate-like B cells of mammals (25, 148). Whether these innate-like B cell-associated proteins detected from our B cell samples are evenly expressed on the surface of all salmon IgM^+^ B cells or only expressed at very high levels on certain sub-populations, is a question that warrants further investigations. This can be determined upon availability of the necessary immunological tools and antibodies to analyze the frequency and level of their surface expression.

Speculations regarding the possible characteristics of salmon IgM B cell populations based solely on pre-conceived notions of the surface phenotype, function, and anatomical distribution of their mammalian counterparts should come with a caveat. Indeed, the conservation of protein functions between mammals and teleosts for many of the B cell-associated CD molecules identified in this study has to be established. Therefore, subsets comprising the salmon IgM^+^ B cells need to be better defined, in order to facilitate further functional studies of these individual surface proteins.

## Summary and Conclusion

In summary, our salmon B cell proteomics approach provide novel information on the surface phenotype of salmon IgM B cells as well as some evolutionary insights in reference to their mammalian counterparts. While possible exclusion of several glycosylated and/or less abundant proteins cannot be discounted due to technical limitations of the protocols used, our detection of 21 CD molecules remains a significant advancement in profiling the salmon B cell surface proteome. In addition, identification of CD22 and CD79A as potential pan-B cell markers represents a considerable positive step toward salmon B cell marker development. Further investigation and evaluation of the salmon CD molecules identified in this study would improve our understanding of B cell dynamics in salmon in the presence of TI and TD antigens during immunostimulation, pathogen infection, or vaccination.

## Ethics Statement

The authors confirm that the experimental protocols used for the live fish experiments were based on the Animal Welfare Act (https://www.regjeringen.no/en/dokumenter/animal-welfare-act/id571188/) and performed in accordance with relevant guidelines and regulations given by the Norwegian Animal Research Authority.

## Author Contributions

MP performed most of the experimental work (leukocyte isolation, FACS sorting, surface protein isolation, qPCR analyses) and wrote the manuscript. J-AB was responsible for the mass spectrometry analyses. LT performed RNA extraction and cDNA syntheses. IJ and JJ collaborated in obtaining funding, helped designed the experiments, and reviewed the manuscript.

### Conflict of Interest Statement

The authors declare that the research was conducted in the absence of any commercial or financial relationships that could be construed as a potential conflict of interest.
