# Supplementary material for: Profiling the Atlantic Salmon IgM+ B Cell Surface Proteome: Novel Information on Teleost Fish B Cell Protein Repertoire and Identification of Potential B Cell Markers
Source: Front Immunol. 2019 Jan 29;10:37. doi: 10.3389/fimmu.2019.00037 (PMC6362898; doi:10.3389/fimmu.2019.00037)
Supplement: Supplementary file 1 [file Table_1.DOCX]

***Table 1.*** Paralogs and isoforms of putative CD molecules in Atlantic salmon genome^a^

| **CD Proteins** | **Chromosome** | **Description** | **Gene Accession No.** | **mRNA length (bp)** | **Protein Product Name** | **Protein Accession No.** | **Amino Acid length** |
| --- | --- | --- | --- | --- | --- | --- | --- |
| **CD9** | **ssa03** | PREDICTED: Salmo salar CD9 molecule (cd9), transcript variant X1 | XM_014193133 | 2276 | PREDICTED: CD9 antigen isoform X1 [Salmo salar] | XP_014048608 | 230 |
|  |  | PREDICTED: Salmo salar CD9 molecule (cd9), transcript variant X2 | XM_014193134 | 2238 | PREDICTED: CD9 antigen isoform X1 [Salmo salar] | XP_014048609 | 230 |
|  |  | PREDICTED: Salmo salar CD9 molecule (cd9), transcript variant X3 | XM_014193135 | 2175 | PREDICTED: CD9 antigen isoform X2 [Salmo salar] | XP_014048610 | 200 |
|  | **ssa06** | PREDICTED: Salmo salar CD9 antigen-like (LOC106607104), mRNA | XM_014203707 | 3005 | PREDICTED: CD9 antigen-like [Salmo salar] | XP_014059182 | 262 |
|  | **ssa10** | PREDICTED: Salmo salar CD9 antigen-like (LOC106561241), transcript variant X1, mRNA | XM_014124968 | 2306 | PREDICTED: CD9 antigen-like isoform X1 [Salmo salar] | XP_013980443 | 228 |
|  |  | PREDICTED: Salmo salar CD9 antigen-like (LOC106561241), transcript variant X2, mRNA | XM_014124969 | 2008 | PREDICTED: CD9 antigen-like isoform X2 [Salmo salar] | XP_013980444 | 227 |
|  | **ssa12** | PREDICTED: Salmo salar CD9 antigen-like (LOC106566039), transcript variant X1, mRNA | XM_014133846 | 2469 | PREDICTED: CD9 antigen-like [Salmo salar] | XP_013989321 | 218 |
|  |  | PREDICTED: Salmo salar CD9 antigen-like (LOC106566039), transcript variant X2, mRNA | XM_014133847 | 2167 | PREDICTED: CD9 antigen-like [Salmo salar] | XP_013989322 | 218 |
|  | **ssa15** | PREDICTED: Salmo salar CD9 antigen-like (LOC106572998), mRNA | XM_014147593 | 935 | PREDICTED: CD9 antigen-like [Salmo salar] | XP_014003068 | 212 |
|  | **ssa16** | PREDICTED: Salmo salar CD9 antigen-like (LOC106573420), transcript variant X1, mRNA | XM_014148450 | 2513 | PREDICTED: CD9 antigen-like isoform X1 [Salmo salar] | XP_014003925 | 227 |
|  |  | PREDICTED: Salmo salar CD9 antigen-like (LOC106573420), transcript variant X2, mRNA | XM_014148451 | 2162 | PREDICTED: CD9 antigen-like isoform X2 [Salmo salar] | XP_014003926 | 226 |
|  | **ssa22** | PREDICTED: Salmo salar CD9 antigen-like (LOC106582727), mRNA | XM_014166099 | 2283 | PREDICTED: CD9 antigen-like [Salmo salar] | XP_014021574 | 217 |
|  | **Unknown** | PREDICTED: Salmo salar CD9 antigen-like (LOC106595881), mRNA | XM_014187224 | 777 | PREDICTED: CD9 antigen-like [Salmo salar] | XP_014042699 | 227 |
| **CD11a** | **ssa02** | PREDICTED: Salmo salar integrin alpha-L-like (LOC106588086), transcript variant X1, mRNA | XM_014176753 | 4892 | PREDICTED: integrin alpha-L-like isoform X1 [Salmo salar] | XP_014032228 | 1196 |
|  |  | PREDICTED: Salmo salar integrin alpha-L-like (LOC106588086), transcript variant X2, mRNA | XM_014176754 | 4860 | PREDICTED: integrin alpha-L-like isoform X2 [Salmo salar] | XP_014032229 | 1187 |
|  |  | PREDICTED: Salmo salar integrin alpha-L-like (LOC106588084), mRNA | XM_014176752 | 5244 | PREDICTED: integrin alpha-L-like [Salmo salar] | XP_014032227 | 1132 |
|  | **ssa12** | PREDICTED: Salmo salar integrin alpha-L-like (LOC106564936), mRNA | XM_014131471 | 3982 | PREDICTED: LOW QUALITY PROTEIN: integrin alpha-L-like [Salmo salar] | XP_013986946 | 1208 |
|  | **Unknown** | PREDICTED: Salmo salar integrin alpha-L-like (LOC106596651), mRNA | XM_014187925 | 870 | PREDICTED: integrin alpha-L-like [Salmo salar] | XP_014043400 | 237 |
| **CD11c** | **ssa04** | PREDICTED: Salmo salar integrin alpha-X-like (LOC106602819), transcript variant X1, mRNA | XM_014195711 | 4519 | PREDICTED: integrin alpha-X-like isoform X1 [Salmo salar] | XP_014051186 | 1159 |
|  |  | PREDICTED: Salmo salar integrin alpha-X-like (LOC106602819), transcript variant X2, mRNA | XM_014195712 | 4516 | PREDICTED: integrin alpha-X-like isoform X2 [Salmo salar] | XP_014051187 | 1158 |
|  | **ssa12** | PREDICTED: Salmo salar integrin alpha-X-like (LOC106564975), mRNA | XM_014131529 | 3722 | PREDICTED: integrin alpha-X-like [Salmo salar] | XP_013987004 | 958 |
|  | **ssa19** | PREDICTED: Salmo salar integrin alpha-X-like (LOC106579408), mRNA | XM_014159297 | 4695 | PREDICTED: integrin alpha-X-like [Salmo salar] | XP_014014772 | 1142 |
| **CD18** | **ssa21** | PREDICTED: Salmo salar integrin beta-2-like (LOC106581859), mRNA | XM_014164283 | 3349 | PREDICTED: integrin beta-2-like [Salmo salar] | XP_014019758 | 785 |
|  | **ssa25** | Salmo salar Integrin beta-2 (itb2), mRNA | NM_001165324 | 3487 | Integrin beta-2 precursor [Salmo salar] | NP_001158796 | 777 |
| **CD22** | **ssa02** | PREDICTED: Salmo salar B-cell receptor CD22-like (LOC106578359), transcript variant X1, mRNA | XM_014157075 | 1086 | PREDICTED: B-cell receptor CD22-like isoform X1 [Salmo salar] | XP_014012550 | 278 |
|  |  | PREDICTED: Salmo salar B-cell receptor CD22-like (LOC106578359), transcript variant X2, mRNA | XM_014157083 | 1454 | PREDICTED: B-cell receptor CD22-like isoform X2 [Salmo salar] | XP_014012558 | 230 |
|  |  | PREDICTED: Salmo salar B-cell receptor CD22-like (LOC106578359), transcript variant X3, mRNA | XM_014157086 | 1366 | PREDICTED: B-cell receptor CD22-like isoform X2 [Salmo salar] | XP_014012561 | 230 |
|  |  | PREDICTED: Salmo salar B-cell receptor CD22-like (LOC106578359), transcript variant X4, mRNA | XM_014157093 | 830 | PREDICTED: B-cell receptor CD22-like isoform X3 [Salmo salar] | XP_014012568 | 229 |
|  |  | PREDICTED: Salmo salar B-cell receptor CD22-like (LOC106578359), transcript variant X5, mRNA | XM_014157102 | 774 | PREDICTED: B-cell receptor CD22-like isoform X4 [Salmo salar] | XP_014012577 | 205 |
|  |  | PREDICTED: Salmo salar B-cell receptor CD22-like (LOC106582456), mRNA | XM_014165590 | 3403 | PREDICTED: B-cell receptor CD22-like [Salmo salar] | XP_014021065 | 1099 |
|  |  | PREDICTED: Salmo salar B-cell receptor CD22-like (LOC106578256), transcript variant X1, mRNA | XM_014156918 | 1906 | PREDICTED: B-cell receptor CD22-like isoform X1 [Salmo salar] | XP_014012393 | 379 |
|  |  | PREDICTED: Salmo salar B-cell receptor CD22-like (LOC106578256), transcript variant X2, mRNA | XM_014156925 | 1898 | PREDICTED: B-cell receptor CD22-like isoform X2 [Salmo salar] | XP_014012400 | 315 |
|  |  | PREDICTED: Salmo salar B-cell receptor CD22-like (LOC106591409), mRNA | XM_014182631 | 1089 | PREDICTED: B-cell receptor CD22-like [Salmo salar] | XP_014038106 | 220 |
|  |  | PREDICTED: Salmo salar B-cell receptor CD22-like (LOC106591373), mRNA | XM_014182597 | 795 | PREDICTED: B-cell receptor CD22-like [Salmo salar] | XP_014038072 | 264 |
|  |  | PREDICTED: Salmo salar B-cell receptor CD22-like (LOC106591359), mRNA | XM_014182585 | 1216 | PREDICTED: B-cell receptor CD22-like [Salmo salar] | XP_014038060 | 223 |
|  |  | PREDICTED: Salmo salar B-cell receptor CD22-like (LOC106591235), mRNA | XM_014182453 | 1982 | PREDICTED: B-cell receptor CD22-like [Salmo salar] | XP_014037928 | 588 |
|  |  | PREDICTED: Salmo salar B-cell receptor CD22-like (LOC106578340), mRNA | XM_014157038 | 2619 | PREDICTED: B-cell receptor CD22-like [Salmo salar] | XP_014012513 | 534 |
|  |  | PREDICTED: Salmo salar B-cell receptor CD22-like (LOC106578274), mRNA | XM_014156944 | 2737 | PREDICTED: B-cell receptor CD22-like [Salmo salar] | XP_014012419 | 637 |
|  |  | PREDICTED: Salmo salar B-cell receptor CD22-like (LOC106578249), mRNA | XM_014156906 | 2070 | PREDICTED: B-cell receptor CD22-like [Salmo salar] | XP_014012381 | 488 |
|  | **ssa03** | PREDICTED: Salmo salar B-cell receptor CD22-like (LOC106601628), mRNA | XM_014193970 | 733 | PREDICTED: B-cell receptor CD22-like [Salmo salar] | XP_014049445 | 234 |
|  |  | PREDICTED: Salmo salar B-cell receptor CD22-like (LOC106601627), mRNA | XM_014193967 | 1247 | PREDICTED: B-cell receptor CD22-like [Salmo salar] | XP_014049442 | 370 |
|  |  | PREDICTED: Salmo salar B-cell receptor CD22-like (LOC106601621), mRNA | XM_014193962 | 4902 | PREDICTED: B-cell receptor CD22-like [Salmo salar] | XP_014049437 | 1259 |
|  | **ssa04** | PREDICTED: Salmo salar B-cell receptor CD22-like (LOC106604036), transcript variant X1, mRNA | [XM_014198371](https://www.ncbi.nlm.nih.gov/nuccore/XM_014198371.1) | 2759 | PREDICTED: B-cell receptor CD22-like isoform X1 [Salmo salar] | XP_014053846 | 612 |
|  |  | PREDICTED: Salmo salar B-cell receptor CD22-like (LOC106604036), transcript variant X2, mRNA | [XM_014198372](https://www.ncbi.nlm.nih.gov/nuccore/XM_014198372.1) | 2124 | PREDICTED: B-cell receptor CD22-like isoform X2 [Salmo salar] | XP_014053847 | 608 |
|  |  | PREDICTED: Salmo salar B-cell receptor CD22-like (LOC106604036), transcript variant X3, mRNA | [XM_014198373](https://www.ncbi.nlm.nih.gov/nuccore/XM_014198373.1) | 2058 | PREDICTED: B-cell receptor CD22-like isoform X3 [Salmo salar] | XP_014053848 | 605 |
|  |  | PREDICTED: Salmo salar B-cell receptor CD22-like (LOC106604036), transcript variant X4, mRNA | [XM_014198374](https://www.ncbi.nlm.nih.gov/nuccore/XM_014198374.1) | 2629 | PREDICTED: B-cell receptor CD22-like isoform X4 [Salmo salar] | XP_014053849 | 591 |
|  |  | PREDICTED: Salmo salar B-cell receptor CD22-like (LOC106604036), transcript variant X5, mRNA | [XM_014198375](https://www.ncbi.nlm.nih.gov/nuccore/XM_014198375.1) | 2677 | PREDICTED: B-cell receptor CD22-like isoform X5 [Salmo salar] | XP_014053850 | 514 |
|  |  | PREDICTED: Salmo salar B-cell receptor CD22-like (LOC106604036), transcript variant X6, mRNA | [XM_014198376](https://www.ncbi.nlm.nih.gov/nuccore/XM_014198376.1) | 2470 | PREDICTED: B-cell receptor CD22-like isoform X6 [Salmo salar] | XP_014053851 | 514 |
|  | **ssa05** | PREDICTED: Salmo salar B-cell receptor CD22-like (LOC106605635), transcript variant X1, mRNA | XM_014201495 | 1948 | PREDICTED: B-cell receptor CD22-like isoform X1 [Salmo salar] | XP_014056970 | 435 |
|  |  | PREDICTED: Salmo salar B-cell receptor CD22-like (LOC106605635), transcript variant X2, mRNA | XM_014201496 | 1946 | PREDICTED: B-cell receptor CD22-like isoform X2 [Salmo salar] | XP_014056971 | 433 |
|  |  | PREDICTED: Salmo salar B-cell receptor CD22-like (LOC106605635), transcript variant X3, mRNA | XM_014201497 | 1922 | PREDICTED: B-cell receptor CD22-like isoform X3 [Salmo salar] | XP_014056972 | 425 |
|  |  | PREDICTED: Salmo salar B-cell receptor CD22-like (LOC106605911), mRNA | XM_014201908 | 1077 | PREDICTED: B-cell receptor CD22-like [Salmo salar] | XP_014057383 | 319 |
|  |  | PREDICTED: Salmo salar B-cell receptor CD22-like (LOC106605910), mRNA | XM_014201907 | 1092 | PREDICTED: B-cell receptor CD22-like [Salmo salar] | XP_014057382 | 315 |
|  |  | PREDICTED: Salmo salar B-cell receptor CD22-like (LOC106605908), partial mRNA | XM_014201905 | 2264 | PREDICTED: B-cell receptor CD22-like [Salmo salar] | XP_014057380 | 594 |
|  |  | PREDICTED: Salmo salar B-cell receptor CD22-like (LOC106605665), mRNA | XM_014201540 | 2142 | PREDICTED: B-cell receptor CD22-like [Salmo salar] | XP_014057015 | 564 |
|  |  | PREDICTED: Salmo salar B-cell receptor CD22-like (LOC106605583), mRNA | XM_014201391 | 954 | PREDICTED: B-cell receptor CD22-like [Salmo salar] | XP_014056866 | 238 |
|  |  | PREDICTED: Salmo salar B-cell receptor CD22-like (LOC106605162), mRNA | XM_014200537 | 3028 | PREDICTED: B-cell receptor CD22-like [Salmo salar] | XP_014056012 | 926 |
|  |  | PREDICTED: Salmo salar B-cell receptor CD22-like (LOC106605006), mRNA | XM_014200195 | 1221 | PREDICTED: B-cell receptor CD22-like [Salmo salar] | XP_014055670 | 369 |
|  | **ssa06** | PREDICTED: Salmo salar B-cell receptor CD22-like (LOC106606237), transcript variant X1, mRNA | XM_014202385 | 1489 | PREDICTED: B-cell receptor CD22-like [Salmo salar] | XP_014057860 | 149 |
|  | **ssa10** | PREDICTED: Salmo salar B-cell receptor CD22-like (LOC106561584), mRNA | XM_014125688 | 1966 | PREDICTED: B-cell receptor CD22-like [Salmo salar] | XP_013981163 | 628 |
|  | **ssa12** | PREDICTED: Salmo salar B-cell receptor CD22-like (LOC106564327), partial mRNA | XM_014130376 | 534 | PREDICTED: B-cell receptor CD22-like [Salmo salar] | XP_013985851 | 144 |
|  | **ssa13** | PREDICTED: Salmo salar B-cell receptor CD22-like (LOC106566698), transcript variant X1, mRNA | XM_014134993 | 2592 | PREDICTED: B-cell receptor CD22-like isoform X1 [Salmo salar] | XP_013990468 | 660 |
|  |  | PREDICTED: Salmo salar B-cell receptor CD22-like (LOC106566698), transcript variant X2, mRNA | XM_014134994 | 2521 | PREDICTED: B-cell receptor CD22-like isoform X2 [Salmo salar] | XP_013990469 | 584 |
|  | **ssa15** | PREDICTED: Salmo salar B-cell receptor CD22-like (LOC106572572), mRNA | XM_014146871 | 492 | PREDICTED: B-cell receptor CD22-like [Salmo salar] | XP_014002346 | 163 |
|  | **ssa18** | PREDICTED: Salmo salar B-cell receptor CD22-like (LOC106577812), mRNA | XM_014156166 | 3258 | PREDICTED: B-cell receptor CD22-like [Salmo salar] | XP_014011641 | 1025 |
|  | **Unknown** | PREDICTED: Salmo salar B-cell receptor CD22-like (LOC106598954), mRNA | XM_014189984 | 1105 | PREDICTED: B-cell receptor CD22-like [Salmo salar] | XP_014045459 | 291 |
|  |  | PREDICTED: Salmo salar B-cell receptor CD22-like (LOC106598058), mRNA | XM_014189143 | 1017 | PREDICTED: B-cell receptor CD22-like [Salmo salar] | XP_014044618 | 241 |
|  |  | PREDICTED: Salmo salar B-cell receptor CD22-like (LOC106596560), partial mRNA | XM_014187839 | 985 | PREDICTED: B-cell receptor CD22-like [Salmo salar] | XP_014043314 | 304 |
|  |  | PREDICTED: Salmo salar B-cell receptor CD22-like (LOC106596279), mRNA | XM_014187586 | 2473 | PREDICTED: B-cell receptor CD22-like [Salmo salar] | XP_014043061 | 436 |
|  |  | PREDICTED: Salmo salar B-cell receptor CD22-like (LOC106596103), mRNA | XM_014187425 | 2706 | PREDICTED: B-cell receptor CD22-like [Salmo salar] | XP_014042900 | 704 |
|  |  | PREDICTED: Salmo salar B-cell receptor CD22-like (LOC106595102), partial mRNA | XM_014186477 | 985 | PREDICTED: B-cell receptor CD22-like [Salmo salar] | XP_014041952 | 304 |
|  |  | PREDICTED: Salmo salar B-cell receptor CD22-like (LOC106593527), mRNA | XM_014184867 | 441 | PREDICTED: B-cell receptor CD22-like [Salmo salar] | XP_014040342 | 146 |
|  |  | PREDICTED: Salmo salar B-cell receptor CD22-like (LOC106592615), mRNA | XM_014183954 | 1512 | PREDICTED: B-cell receptor CD22-like [Salmo salar] | XP_014039429 | 478 |
| **CD40** | **ssa15** | Salmo salar Tumor necrosis factor receptor superfamily member 5 (tnr5), mRNA | NM_001141236 | 1533 | Tumor necrosis factor receptor superfamily member 5 precursor [Salmo salar] | NP_001134708 | 354 |
|  | **ssa13** | PREDICTED: Salmo salar tumor necrosis factor receptor superfamily member 5-like (LOC106567123), mRNA | XM_014136048 | 2370 | PREDICTED: tumor necrosis factor receptor superfamily member 5-like [Salmo salar] | XP_013991523 | 317 |
| **CD45** | **ssa10** | PREDICTED: Salmo salar protein tyrosine phosphatase, receptor type, C (ptprc), transcript variant X1, mRNA | XM_014123566 | 5076 | PREDICTED: receptor-type tyrosine-protein phosphatase C isoform X1 [Salmo salar] | XP_013979041 | 1412 |
|  |  | PREDICTED: Salmo salar protein tyrosine phosphatase, receptor type, C (ptprc), transcript variant X2, mRNA | XM_014123567 | 5132 | PREDICTED: receptor-type tyrosine-protein phosphatase C isoform X1 [Salmo salar] | XP_013979042 | 1412 |
|  |  | PREDICTED: Salmo salar protein tyrosine phosphatase, receptor type, C (ptprc), transcript variant X3, mRNA | XM_014123568 | 4950 | PREDICTED: receptor-type tyrosine-protein phosphatase C isoform X2 [Salmo salar] | XP_013979043 | 1370 |
|  |  | PREDICTED: Salmo salar protein tyrosine phosphatase, receptor type, C (ptprc), transcript variant X4, mRNA | XM_014123569 | 4947 | PREDICTED: receptor-type tyrosine-protein phosphatase C isoform X3 [Salmo salar] | XP_013979044 | 1369 |
|  |  | PREDICTED: Salmo salar protein tyrosine phosphatase, receptor type, C (ptprc), transcript variant X5, mRNA | XM_014123570 | 4661 | PREDICTED: receptor-type tyrosine-protein phosphatase C isoform X4 [Salmo salar] | XP_013979045 | 1274 |
|  |  | PREDICTED: Salmo salar protein tyrosine phosphatase, receptor type, C (ptprc), transcript variant X6, mRNA | XM_014123571 | 4532 | PREDICTED: receptor-type tyrosine-protein phosphatase C isoform X5 [Salmo salar] | XP_013979046 | 1231 |
|  |  | PREDICTED: Salmo salar protein tyrosine phosphatase, receptor type, C (ptprc), transcript variant X7, mRNA | XM_014123572 | 4469 | PREDICTED: receptor-type tyrosine-protein phosphatase C isoform X6 [Salmo salar] | XP_013979047 | 1210 |
| **CD53** | **ssa20** | PREDICTED: Salmo salar leukocyte surface antigen CD53-like (LOC106580528), transcript variant X1, mRNA | XM_014161700 | 1586 | PREDICTED: leukocyte surface antigen CD53-like isoform X1 [Salmo salar] | XP_014017175 | 229 |
|  |  | PREDICTED: Salmo salar leukocyte surface antigen CD53-like (LOC106580528), transcript variant X2, mRNA | XM_014161701 | 1499 | PREDICTED: leukocyte surface antigen CD53-like isoform X1 [Salmo salar] | XP_014017176 | 229 |
|  |  | PREDICTED: Salmo salar leukocyte surface antigen CD53-like (LOC106580528), transcript variant X3, mRNA | XM_014161702 | 1425 | PREDICTED: leukocyte surface antigen CD53-like isoform X2 [Salmo salar] | XP_014017177 | 212 |
|  |  | PREDICTED: Salmo salar leukocyte surface antigen CD53-like (LOC106580528), transcript variant X4, mRNA | XM_014161703 | 1493 | PREDICTED: leukocyte surface antigen CD53-like isoform X3 [Salmo salar] | XP_014017178 | 198 |
|  |  | PREDICTED: Salmo salar leukocyte surface antigen CD53-like (LOC106580528), transcript variant X5, mRNA | XM_014161704 | 1496 | PREDICTED: leukocyte surface antigen CD53-like isoform X4 [Salmo salar] | XP_014017179 | 196 |
|  |  | PREDICTED: Salmo salar leukocyte surface antigen CD53-like (LOC106580528), transcript variant X6, mRNA | XM_014161705 | 1517 | PREDICTED: leukocyte surface antigen CD53-like isoform X4 [Salmo salar] | XP_014017180 | 196 |
|  |  | PREDICTED: Salmo salar leukocyte surface antigen CD53-like (LOC106580528), transcript variant X7, mRNA | XM_014161706 | 1469 | PREDICTED: leukocyte surface antigen CD53-like isoform X4 [Salmo salar] | XP_014017181 | 196 |
|  | **ssa24** | Salmo salar Leukocyte surface antigen CD53 (cd53), mRNA | NM_001140576 | 1178 | Leukocyte surface antigen CD53 [Salmo salar] | NP_001134048 | 234 |
|  |  | PREDICTED: Salmo salar leukocyte surface antigen CD53-like (LOC106585137), mRNA | XM_014171005 | 1379 | PREDICTED: leukocyte surface antigen CD53-like [Salmo salar] | XP_014026480 | 232 |
| **CD63** | **ssa03** | PREDICTED: Salmo salar CD63 antigen-like (LOC106600980), mRNA | XM_014192798 | 588 | PREDICTED: CD63 antigen-like [Salmo salar] | XP_014048273 | 195 |
|  | **ssa13** | PREDICTED: Salmo salar CD63 antigen-like (LOC106567183), transcript variant X1, mRNA | XM_014136194 | 1271 | PREDICTED: CD63 antigen-like [Salmo salar] | XP_013991669 | 241 |
|  |  | PREDICTED: Salmo salar CD63 antigen-like (LOC106567183), transcript variant X2, mRNA | XM_014136195 | 1240 | PREDICTED: CD63 antigen-like [Salmo salar] | XP_013991670 | 241 |
|  | **ssa14** | PREDICTED: Salmo salar CD63 antigen-like (LOC106569561), mRNA | XM_014141035 | 1364 | PREDICTED: CD63 antigen-like [Salmo salar] | XP_013996510 | 369 |
|  | **ssa15** | Salmo salar Cd63 antigen (cd63), mRNA | NM_001140602 | 1378 | CD63 antigen [Salmo salar] | NP_001134074 | 241 |
| **CD68** | **ssa07** | Salmo salar Macrosialin (cd68), mRNA | NM_001165385 | 3323 | Macrosialin precursor [Salmo salar] | NP_001158857 | 355 |
| **CD79A** | **ssa02** | PREDICTED: Salmo salar B-cell antigen receptor complex-associated protein alpha chain-like (LOC106577375), transcript variant X1, mRNA | XM_014155344 | 1033 | PREDICTED: B-cell antigen receptor complex-associated protein alpha chain-like isoform X1 [Salmo salar] | XP_014010819 | 248 |
|  |  | PREDICTED: Salmo salar B-cell antigen receptor complex-associated protein alpha chain-like (LOC106577375), transcript variant X2, mRNA | XM_014155349 | 1030 | PREDICTED: B-cell antigen receptor complex-associated protein alpha chain-like isoform X2 [Salmo salar] | XP_014010824 | 247 |
|  | **ssa05** | PREDICTED: Salmo salar B-cell antigen receptor complex-associated protein alpha chain-like (LOC106605673), transcript variant X1, mRNA | XM_014201561 | 1267 | PREDICTED: B-cell antigen receptor complex-associated protein alpha chain-like isoform X1 [Salmo salar] | XP_014057036 | 234 |
|  |  | PREDICTED: Salmo salar B-cell antigen receptor complex-associated protein alpha chain-like (LOC106605673), transcript variant X2, mRNA | XM_014201562 | 1264 | PREDICTED: B-cell antigen receptor complex-associated protein alpha chain-like isoform X2 [Salmo salar] | XP_014057037 | 233 |
|  | **Unknown** | PREDICTED: Salmo salar B-cell antigen receptor complex-associated protein alpha chain-like (LOC106596637), transcript variant X1, mRNA | XM_014187907 | 964 | PREDICTED: B-cell antigen receptor complex-associated protein alpha chain-like isoform X1 [Salmo salar] | XP_014043382 | 234 |
|  |  | PREDICTED: Salmo salar B-cell antigen receptor complex-associated protein alpha chain-like (LOC106596637), transcript variant X2, mRNA | XM_014187908 | 961 | PREDICTED: B-cell antigen receptor complex-associated protein alpha chain-like isoform X2 [Salmo salar] | XP_014010824 | 247 |
|  |  | PREDICTED: Salmo salar B-cell antigen receptor complex-associated protein alpha chain-like (LOC106595045), transcript variant X1, mRNA | XM_014186423 | 964 | PREDICTED: B-cell antigen receptor complex-associated protein alpha chain-like isoform X1 [Salmo salar] | XP_014041898 | 234 |
|  |  | PREDICTED: Salmo salar B-cell antigen receptor complex-associated protein alpha chain-like (LOC106595045), transcript variant X2, mRNA | XM_014186424 | 961 | PREDICTED: B-cell antigen receptor complex-associated protein alpha chain-like isoform X2 [Salmo salar] | XP_014041899 | 233 |
| **CD81** | **ssa10** | PREDICTED: Salmo salar CD81 antigen-like (LOC106561812), mRNA | XM_014126059 | 1209 | PREDICTED: CD81 antigen-like [Salmo salar] | XP_013981534 | 239 |
|  | **ssa11** | PREDICTED: Salmo salar CD81 antigen-like (LOC106563533), mRNA | XM_014129215 | 3459 | PREDICTED: CD81 antigen-like [Salmo salar] | XP_013984690 | 241 |
|  | **ssa23** | PREDICTED: Salmo salar CD81 antigen-like (LOC106584765), mRNA | XM_014170313 | 1140 | PREDICTED: CD81 antigen-like [Salmo salar] | XP_014025788 | 239 |
|  | **ssa26** | PREDICTED: Salmo salar CD81 antigen-like (LOC106587471), mRNA | XM_014175885 | 3372 | PREDICTED: CD81 antigen-like [Salmo salar] | XP_014031360 | 241 |
| **CD84-H1** | **ssa07** | PREDICTED: Salmo salar SLAM family member 9-like (LOC106608989), mRNA | XM_014207304 | 1517 | PREDICTED: SLAM family member 9-like [Salmo salar] | XP_014062779 | 381 |
| **CD87** | **ssa02** | PREDICTED: Salmo salar urokinase plasminogen activator surface receptor-like (LOC106578012), mRNA | XM_014156544 | 1737 | PREDICTED: urokinase plasminogen activator surface receptor-like [Salmo salar] | XP_014012019 | 228 |
|  |  | PREDICTED: Salmo salar urokinase plasminogen activator surface receptor-like (LOC106578009), mRNA | XM_014156531 | 1316 | PREDICTED: urokinase plasminogen activator surface receptor-like [Salmo salar] | XP_014012006 | 205 |
|  | **ssa05** | PREDICTED: Salmo salar urokinase plasminogen activator surface receptor-like (LOC106605607), mRNA | XM_014201441 | 1338 | PREDICTED: urokinase plasminogen activator surface receptor-like [Salmo salar] | XP_014056916 | 205 |
| **CD98** | **ssa04** | PREDICTED: Salmo salar 4F2 cell-surface antigen heavy chain-like (LOC106603161), mRNA | XM_014196469 | 2266 | PREDICTED: 4F2 cell-surface antigen heavy chain-like [Salmo salar] | XP_014051944 | 515 |
|  | **ssa07** | Salmo salar 4F2 cell-surface antigen heavy chain (4f2), mRNA | NM_001140307 | 2156 | 4F2 cell-surface antigen heavy chain [Salmo salar] | NP_001133779 | 510 |
|  | **ssa11** | PREDICTED: Salmo salar solute carrier family 3 (amino acid transporter heavy chain), member 2 (slc3a2), mRNA | XM_014129152 | 2557 | PREDICTED: 4F2 cell-surface antigen heavy chain [Salmo salar] | XP_013984627 | 511 |
|  | **ssa18** | PREDICTED: Salmo salar 4F2 cell-surface antigen heavy chain-like (LOC106578137), transcript variant X1, mRNA | XM_014156756 | 1984 | PREDICTED: 4F2 cell-surface antigen heavy chain-like isoform X1 [Salmo salar] | XP_014012231 | 526 |
|  |  | PREDICTED: Salmo salar 4F2 cell-surface antigen heavy chain-like (LOC106578137), transcript variant X2, mRNA | XM_014156757 | 2006 | PREDICTED: 4F2 cell-surface antigen heavy chain-like isoform X2 [Salmo salar] | XP_014012232 | 526 |
|  |  | PREDICTED: Salmo salar 4F2 cell-surface antigen heavy chain-like (LOC106578137), transcript variant X3, mRNA | XM_014156758 | 1690 | PREDICTED: 4F2 cell-surface antigen heavy chain-like isoform X3 [Salmo salar] | XP_014012233 | 517 |
|  |  | PREDICTED: Salmo salar 4F2 cell-surface antigen heavy chain-like (LOC106578137), transcript variant X4, mRNA | XM_014156759 | 1800 | PREDICTED: 4F2 cell-surface antigen heavy chain-like isoform X4 [Salmo salar] | XP_014012234 | 507 |
|  |  | PREDICTED: Salmo salar 4F2 cell-surface antigen heavy chain-like (LOC106578137), transcript variant X5, mRNA | XM_014156760 | 1803 | PREDICTED: 4F2 cell-surface antigen heavy chain-like isoform X4 [Salmo salar] | XP_014012235 | 507 |
|  |  | PREDICTED: Salmo salar 4F2 cell-surface antigen heavy chain-like (LOC106578137), transcript variant X6, mRNA | XM_014156761 | 1675 | PREDICTED: 4F2 cell-surface antigen heavy chain-like isoform X4 [Salmo salar] | XP_014012236 | 507 |
|  |  | PREDICTED: Salmo salar 4F2 cell-surface antigen heavy chain-like (LOC106578137), transcript variant X7, mRNA | XM_014156762 | 1903 | PREDICTED: 4F2 cell-surface antigen heavy chain-like isoform X4 [Salmo salar] | XP_014012237 | 484 |
| **CD147** | **ssa10** | PREDICTED: Salmo salar basigin (Ok blood group) (bsg), transcript variant X1, mRNA | XM_014216323 | 2469 | PREDICTED: basigin isoform X1 [Salmo salar] | XP_014071798 | 372 |
|  |  | PREDICTED: Salmo salar basigin (Ok blood group) (bsg), transcript variant X2, mRNA | XM_014216324 | 2457 | PREDICTED: basigin isoform X2 [Salmo salar] | XP_014071799 | 368 |
|  |  | PREDICTED: Salmo salar basigin (Ok blood group) (bsg), transcript variant X3, mRNA | XM_014216325 | 2419 | PREDICTED: basigin isoform X3 [Salmo salar] | XP_014071800 | 272 |
| **CD156c** | **ssa10** | PREDICTED: Salmo salar disintegrin and metalloproteinase domain-containing protein 10-like (LOC106561772), partial mRNA | XM_014126009 | 882 | PREDICTED: disintegrin and metalloproteinase domain-containing protein 10-like, partial [Salmo salar] | XP_013981484 | 294 |
|  |  | PREDICTED: Salmo salar disintegrin and metalloproteinase domain-containing protein 10-like (LOC106561774), mRNA | XM_014126011 | 4088 | PREDICTED: disintegrin and metalloproteinase domain-containing protein 10-like [Salmo salar] | XP_013981486 | 515 |
|  | **ssa11** | PREDICTED: Salmo salar ADAM metallopeptidase domain 10 (adam10), | XM_014126901 | 5505 | PREDICTED: disintegrin and metalloproteinase domain-containing protein 10 [Salmo salar] | XP_013982376 | 763 |
|  | **ssa23** | PREDICTED: Salmo salar disintegrin and metalloproteinase domain-containing protein 10-like (LOC106584732), transcript variant X1, mRNA | XM_014170266 | 4557 | PREDICTED: disintegrin and metalloproteinase domain-containing protein 10-like isoform X1 [Salmo salar] | XP_014025741 | 835 |
|  |  | PREDICTED: Salmo salar disintegrin and metalloproteinase domain-containing protein 10-like (LOC106584732), transcript variant X5, mRNA | XM_014170267 | 7285 | PREDICTED: disintegrin and metalloproteinase domain-containing protein 10-like isoform X2 [Salmo salar] | XP_014025742 | 800 |
|  | **ssa26** | PREDICTED: Salmo salar disintegrin and metalloproteinase domain-containing protein 10-like (LOC106587342), mRNA | XM_014175660 | 5439 | PREDICTED: disintegrin and metalloproteinase domain-containing protein 10-like [Salmo salar] | XP_014031135 | 762 |
| **CD184** | **ssa21** | PREDICTED: Salmo salar C-X-C chemokine receptor type 4-like (LOC106581841), mRNA | XM_014164248 | 1674 | PREDICTED: C-X-C chemokine receptor type 4-like [Salmo salar] | XP_014019723 | 356 |
|  | **ssa25** | Salmo salar C-X-C chemokine receptor type 4 (cxcr4), mRNA | NM_001165293 | 1633 | C-X-C chemokine receptor type 4 [Salmo salar] | NP_001158765 | 362 |
| **CD185** | **ssa09** | PREDICTED: Salmo salar C-X-C chemokine receptor type 5-like (LOC106613395), mRNA | XM_014215581 | 2420 | PREDICTED: C-X-C chemokine receptor type 5-like [Salmo salar] | XP_014071056 | 340 |
| **CDw199** | **ssa03** | PREDICTED: Salmo salar C-C chemokine receptor type 9-like (LOC106597311), mRNA | XM_014188520 | 2148 | PREDICTED: C-C chemokine receptor type 9-like [Salmo salar] | XP_014043995 | 368 |
|  | **ssa14** | Salmo salar C-C chemokine receptor type 9 (ccr9), mRNA | NM_001140518 | 2282 | C-C chemokine receptor type 9 [Salmo salar] | NP_001133990 | 368 |
|  | **ssa19** | PREDICTED: Salmo salar C-C chemokine receptor type 9-like (LOC106578656), transcript variant X1, mRNA | XM_014157695 | 1999 | PREDICTED: C-C chemokine receptor type 9-like [Salmo salar] | XP_014013170 | 366 |
|  |  | PREDICTED: Salmo salar C-C chemokine receptor type 9-like (LOC106578656), transcript variant X2, mRNA | XM_014157696 | 1828 | PREDICTED: C-C chemokine receptor type 9-like [Salmo salar] | XP_014013171 | 366 |
|  |  | PREDICTED: Salmo salar C-C chemokine receptor type 9-like (LOC106578656), transcript variant X3, mRNA | XM_014157697 | 2187 | PREDICTED: C-C chemokine receptor type 9-like [Salmo salar] | XP_014013172 | 366 |
| **CD282** | **ssa07** | PREDICTED: Salmo salar toll-like receptor 2 (LOC106608430), transcript variant X1, mRNA | XM_014206364 | 2709 | PREDICTED: toll-like receptor 2 [Salmo salar] | XP_014061839 | 806 |
|  |  | PREDICTED: Salmo salar toll-like receptor 2 (LOC106608430), transcript variant X2, mRNA | XM_014206365 | 2625 | PREDICTED: toll-like receptor 2 [Salmo salar] | XP_014061840 | 806 |

^a^ Sequences obtained from partial and full-length cDNAs or predicted from genome scaffolds by automated computational analysis using gene prediction method: Gnomon (BioProject: [PRJNA287919](https://www.ncbi.nlm.nih.gov/bioproject/PRJNA287919))
